# Case Report: Identification of a novel *CASK* missense variant in a Chinese family with MICPCH

**DOI:** 10.3389/fgene.2022.933785

**Published:** 2022-08-25

**Authors:** Runfeng Zhang, Peng Jia, Yanyi Yao, Feng Zhu

**Affiliations:** ^1^ College of Life Sciences, Hubei Normal University, Huangshi, China; ^2^ Department of Cardiology, Union Hospital, Tongji Medical College, Huazhong University of Science and Technology, Wuhan, China; ^3^ Medical Genetic Center, Maternal and Child Health Hospital of Hubei Province, Wuhan, China; ^4^ Clinic Center of Human Gene Research, Union Hospital, Tongji Medical College, Huazhong University of Science and Technology, Wuhan, China

**Keywords:** *CASK* gene, spinocerebellar ataxia, mutation, whole-exome sequencing, mental retardation

## Abstract

Mental retardation and microcephaly with pontine and cerebellar hypoplasia (MICPCH) is a rare genetic disorder that results in varying levels of pontocerebellar hypoplasia, microcephaly, and severe intellectual disabilities. Prior genetic analyses have identified the *CASK* gene as a driver of MICPCH. Herein, we analyzed a Chinese family with MICPCH. The index patient was an 8-year-old male. He and his 3-year-old brother suffered from microcephaly, pontocerebellar hypoplasia, serious mental retardation, ataxia, gait disorder, and inability to speak. Through a combination of whole-exome sequencing and subsequent Sanger sequencing, a novel X-linked missense mutation, c.1882G>C (p.D628H) in the *CASK* gene, was identified in two siblings, as well as their mother and grandmother, who exhibited mild mental retardation. Other family members with negative genetic testing were normal. *In silico* analyses indicated that this missense mutation was predicted to reduce CASK protein stability, disrupt the SRC homology 3 (SH3) domain, and abolish its function. In summary, we identified a novel missense variate in CASK associated with MICPCH. Our work facilitates the diagnosis of the disease in this family and broadens the gene variant spectrum of the *CASK* in MICPCH patients.

## Introduction

Mental retardation and microcephaly with pontine and cerebellar hypoplasia (MICPCH, MIM 300749) is a rare X-linked genetic condition characterized by varying degrees of pontocerebellar hypoplasia, microcephaly, and severe intellectual disability. Affected patients typically exhibit limited psychomotor developmental progression, including limited speech or ambulation, axial hypotonia, and, in some cases, hypertonia (Moog et al., 2011; Moog et al., 2015). Females are most commonly affected by this condition, presenting with progressive microcephaly and severe physical and intellectual developmental delays such as those outlined above, seizures, and behavioral disorders (Adam et al., 1993). Patients rarely exhibit congenital defects but occasionally develop a distinctive facial phenotype, grow short of stature, have sensorineural hearing loss, and may have a variety of ocular abnormalities (Moog et al., 2011; Burglen et al., 2012). To date, only a small number of male MICPCH patients have been reported (Burglen et al., 2012; Saitsu et al., 2012; Takanashi et al., 2012; Nakamura et al., 2014), owing to the reduced viability and increased *in utero* lethality rates associated with this rare disorder (Najm et al., 2008). Certain familial cases affecting male patients have been described, in which patients present with an intellectual disability ranging from mild to severe with or without nystagmus or are diagnosed with the so-called FG-syndrome (Piluso et al., 2009; Tarpey et al., 2009; Hackett et al., 2010).

The *CASK* gene encoding a calcium/calmodulin-dependent serine protein kinase has been identified as an important candidate associated with the pathogenesis of MICPCH, owing to its critical role during neuronal development and the fact that mice harboring mutations in this gene had smaller brains, an aberrant cranial shape, and a higher prevalence of cleft palates (Najm et al., 2008). The *CASK* gene is located on chromosome Xp11.4 in humans (Dimitratos et al., 1998; Stevenson et al., 2000). In synapses, CASK regulates presynaptic interactions and the formation of the presynaptic termini, maintains postsynaptic dendritic spine morphology, and regulates postsynaptic ion channels (Hsueh, 2009). These CASK functional roles may at least partially account for the brain developmental deficit and intellectual disability observed in patients with *CASK* variants. In individuals with MICPCH or X-linked intellectual disability (XLID), over 100 investigations have documented a variety of *CASK* variants, including missense variants (LaConte et al., 2018), intragenic duplications (Hayashi et al., 2012), splice site variants (Dunn et al., 2017), rearrangements, and deletion-insertion variants (Saitsu et al., 2012; Moog et al., 2015; Hayashi et al., 2017).

Herein, we describe a novel missense variant (c.1882G>C) in *CASK*, which was detected in two male siblings displaying a MICPCH phenotype, as well as in their mother and grandmother, both with mild intellectual disabilities. It was predicted that this variant would decrease the CASK protein stability, disrupt the SRC homology 3 (SH3) domain, and abolish its functional role.

## Case Report

**FIGURE 1:**
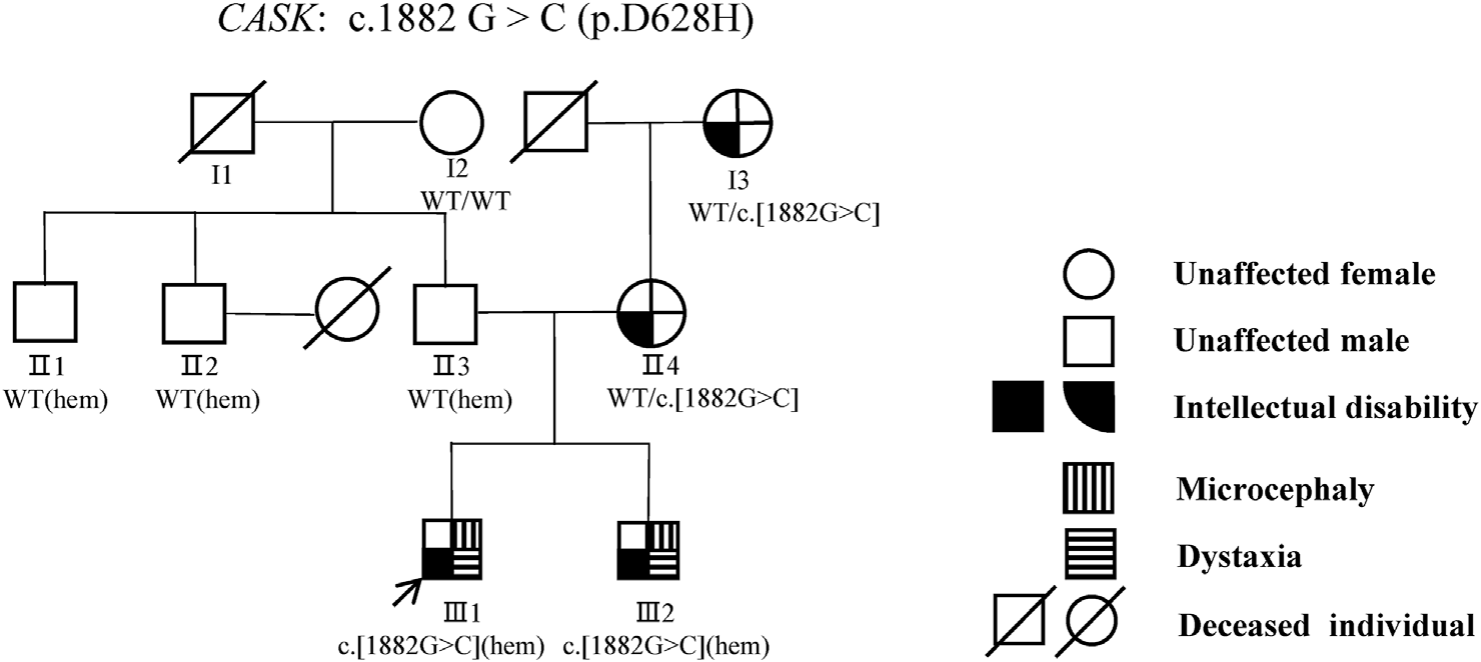
Pedigree of a three-generation family suffering from spinocerebellar ataxia. Males are represented by squares and females by circles. Intellectual disability is indicated by a black square, dystaxia by parallel horizontal lines, and microcephaly by parallel vertical lines. The black arrows indicated the probands (III1) mut+, mutation present; wt, wild type, het, heterozygous; hem, hemizygous.

**FIGURE 2:**
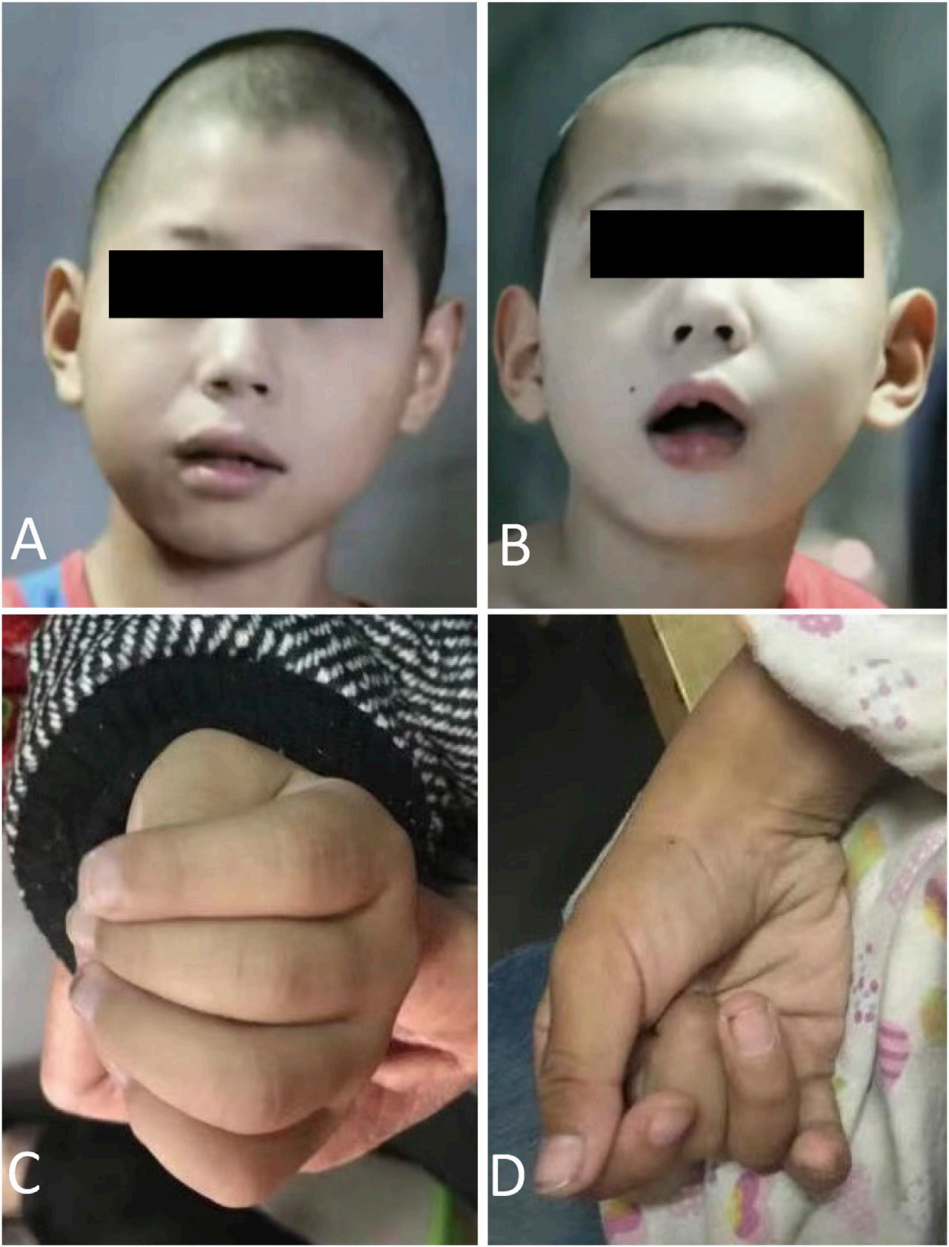
Facial features and involuntary flexion of fingers (III1 and III2).

The proband (III1) was an 8-year-old male who was referred to our medical genetic center by the neuropediatrician for microcephaly, intellectual disability, ataxia, gait disorder, and inability to speak (Figure 1). His perinatal history was unremarkable. His previous medical history included global developmental delay, hearing loss, loss of language, and a seizure, which was managed with carbamazepine therapy. He had microcephaly, oval faces, and large ears (Figures 2A,B). He had motor coordination issues, fine and gross motor delays, and impaired hand function (involuntary flexion of the hands, Figures 2C,D). Independent ambulation was not possible. He was assessed for profound intellectual disability and scored below the first percentile for the Wechsler Children Intelligence Scale (Chinese revision). In addition to exhibiting the same phenotypes, his 3-year-old brother (III2) also has strabismus. The two siblings (III1 and III2) were denied appropriate rehabilitation therapy because of financial limitations. Following a 2-year observation period, the proband’s height and head circumference were, respectively, 1 and 2 standard deviations below the average for Chinese boys of the same age. According to the reassessment of the Wechsler Children Intelligence Scale (Chinese revision), he still had a profound intellectual disability.

**FIGURE 3:**
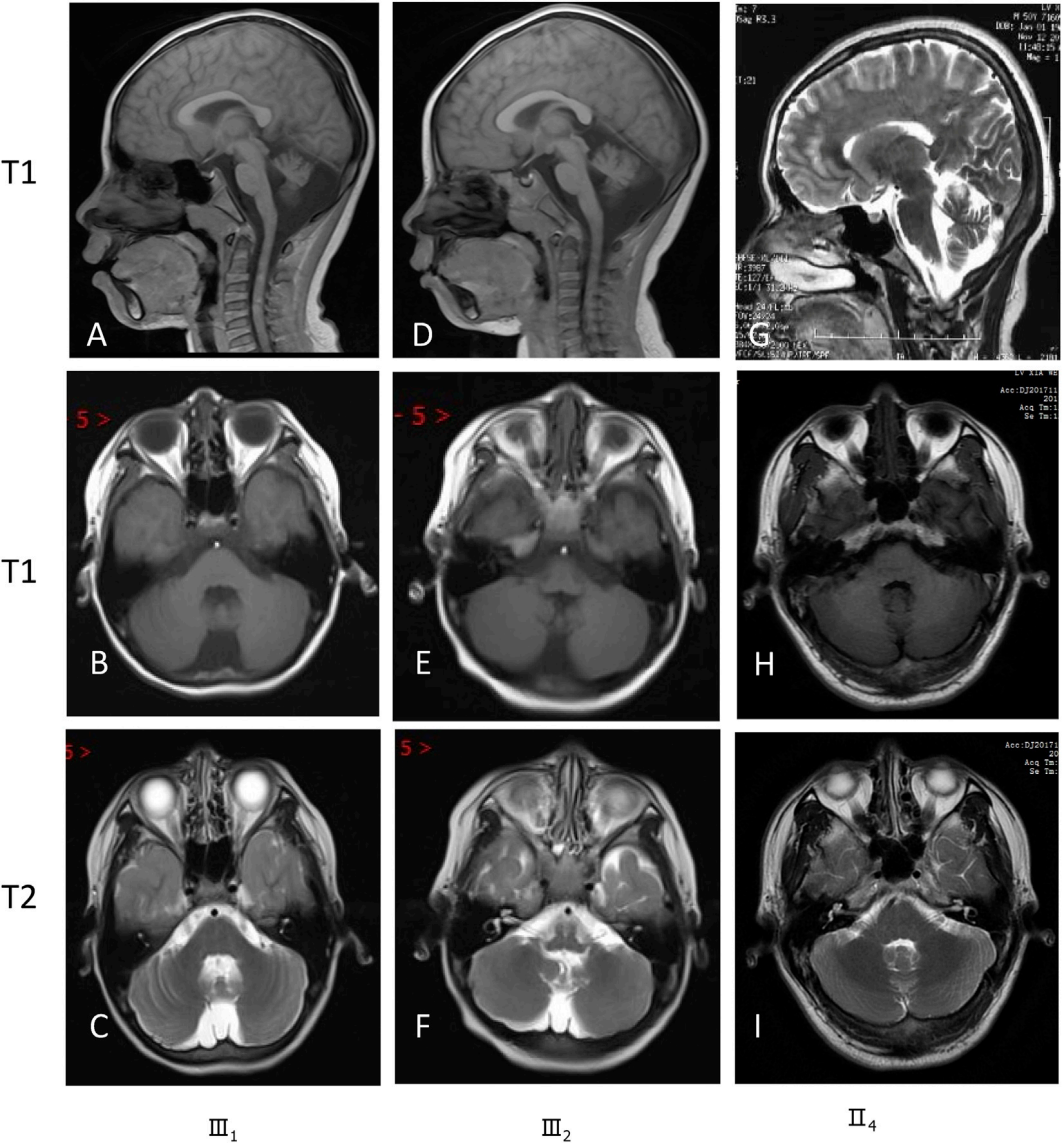
Magnetic resonance images show severe hypoplasia of the cerebellum (III1 and III2).

Brain magnetic resonance imaging (MRI) revealed hypoplasia in both cerebellar hemispheres and the vermis in these siblings (III1 and III2) (Figure 3). Pedigree analysis revealed that the proband’s mother (II4) and grandmother (I3) were similarly affected with mild intellectual disability (Figure 1). According to the assessment results of the Wechsler Adult Intelligence Scale (Chinese revision), I3 and II4 had mild and moderate intellectual disability, respectively. Clinical features of these studied family members are compiled in Supplementary Table S1. Brain MRI of their parents (II3 and II4) exhibited unaffected cerebellar structures (Figures 3G–I).

As copy number variations (CNVs) are an important genetic cause of intellectual disability, an SNP array was performed on the proband. Genomic DNA was extracted from blood cells by Qiagen DNA Blood mini kit following the manufacturer’s protocol. SNP array analysis was performed with CytoScan 750K array (Affymetrix, Santa Clara, CA, United States), including 550,000 CNVs probes and 200,000 SNP probes, according to the manufacturer’s instructions. The thresholds for our detection criteria for CNVs were set at ≥200 kb for gains, ≥100 kb for losses, and ≥10 Mb for the loss of heterozygosity (LOH). A 208 kb deletion on chromosome 11p12, arr[GRCh37] 11p12 (40,658,001-40,866,121)x1, was detected in the proband (III1) and his mother (III4), but not in his brother (III2) or his father (II3). This deletion affects a single gene, *LRRC4C*. According to the DECIPHER database (https://www.deciphergenomics.org/), which compares patient genotypic and phenotypic data, this structural variation in the *LRRC4C* gene was predicted to be likely benign, suggesting this microdeletion is unlikely related to the observed phenotypes in this study. In order to exclude spinocerebellar ataxia (SCA)1, SCA2, and SCA3, the SCA repeat expansion panel was conducted in the proband (III1), which was found not abnormal (Supplementary Table S2). Finally, we performed whole-exome sequencing (WES) on the proband (III1), his brother (III2), and his parents (II3 and II4) to detect disease-related SNVs and Indels.

Based on the aligned reads from WES data, 71,106 initial variants (62,222 SNVs, 3,313 indels) were identified in the proband. Through our filter strategy-based analysis pipeline in-house (Xiong et al., 2019), 601 variants were kept. Patients in the family had distinct gender disparities in their disease phenotypes; although female patients had mild mental retardation, male patients had more severe disease phenotypes, suggesting X-linked disorders. Eight variants from eight genes (*ZRSR2*, *DCAF8L2*, *CASK, AKAP4*, *AR*, *ATP7A*, *NXF2*, and *KIAA1210*) in the X chromosome were kept after the filter of X-linked inheritance (Supplementary Table S3). Among these variants, a novel c.1882G>C variant in the *CASK* gene (NM_003688.3), which results in an Asp-to-His amino acid change at codon 628 (p.D628H), is the most likely candidate variant for the MICPCH phenotype in this family. Subsequent Sanger sequencing confirmed that two affected males (III1, III2) were hemizygous and two females (I3, II4) with mild symptoms were heterozygous, whereas all unaffected family members were wild-type (I2, II2, III3) (Supplementary Figure S1). This variant co-segregated with the disease in the family and was not found in the Genome Aggregation Database, 1000 Genomes, Exome Aggregation Consortium database, and our in-house database of 100 healthy Chinese adults. Comparative amino acid sequence alignment of *CASK* across various species showed the aspartic acid at codon 628 was highly conserved (Supplementary Figure S2).

**FIGURE 4:**
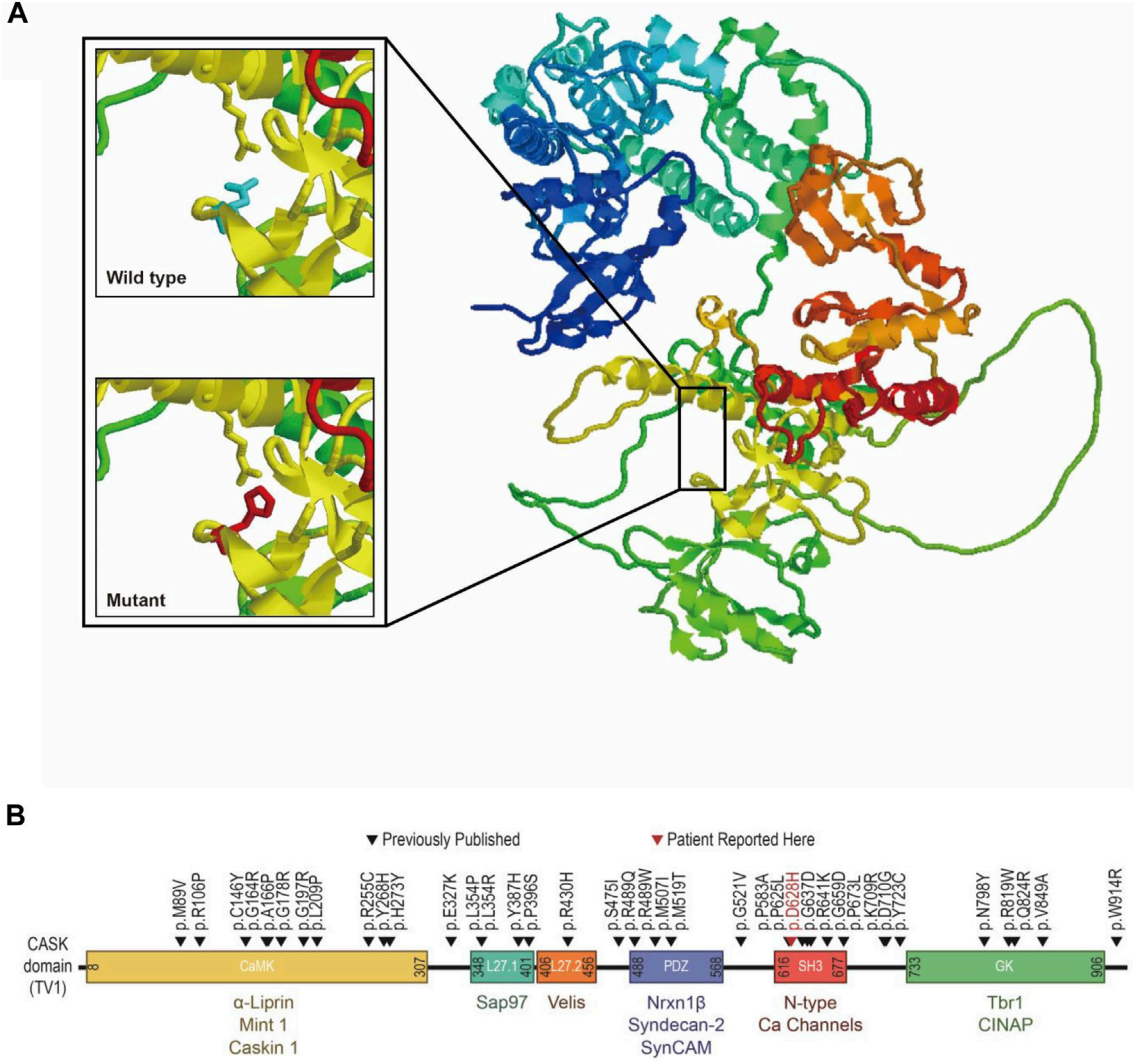
Structural prediction and position mapping of D628H of CASK protein. **(A)** Structural prediction of wild-type D and the mutant H residues at the 628° position. Bold, the wild-type and mutant side chain are shown in blue and red, respectively. **(B)** Position mapping of previously reported missense mutations p.D628Hin this study, CASK protein.

CASK protein structure (AF-O14936-F1-model_V2) was downloaded from AlphaFold Protein Structure Database (https://www.alphafold.ebi.ac.uk) as the template. By entering protein sequences into the SWISS-MODEL website (https://swissmodel.expasy.org) and then visualizing with RasMol (http://www.rasmol.org), structural models of the wild-type and mutant CASK proteins were created (Figure 4). By contrasting the interactions of the relevant amino acids and, consequently, the impact of protein structure, it was possible to determine the probable pathogenic implications of the particular variant. According to molecular dynamics simulations, the *CASK* p.D628H variant within the CASK SH3 domain introduces a neutrally charged histidine that is bigger than negatively charged aspartic acid. In the wild-type structure, Asp628 is located in the folder structure of two prolines (amino sequences of 625∼632: PAKDDLIP), with a side chain toward the α-helix in which Arg681 resided. Salt-bridge formation may occur between the Arg681 and Asp628 because they are at a distance of 2.71Å in this protein. The fact that Asp628 is conserved across many species of CASK protein suggests that the Arg-Asp salt bridge may play an important role in the formation and operation of CASK protein. However, the predicted Arg-Asp salt bridge will be broken by replacing the negatively charged aspartic acid with the non-polar amino acid histidine, potentially disrupting the domain and the interaction between CASK and neurexin (Figure 4). Moreover, the overall stability of the resultant protein was additionally predicted to be reduced (ΔΔG_pred_ = −1.12937 by Mupro and −1.39 by SAAFEC-SEQ) (Cheng et al., 2005; Li et al., 2021). PolyPhen-2 (Adzhubei et al., 2013), PROVEAN (Choi and Chan, 2015), and SIFT tools predicted this variant to be deleterious (Ng and Henikoff, 2003). According to ACMG/AMP guidelines (Richards et al., 2015), the *CASK* p.D628H was classified as likely pathogenic (PM2+PP1+PP2+PP3+PP4).

Because all filtering tools produce false negatives, the known pathogenic variants related to microcephaly and/or pontocerebellar hypoplasia may be ruled out following our filtering process. To identify the known pathogenic variants that might be excluded, we generated a list containing the variants in 78 known disease-causing genes that might cause the phenotypes of microcephaly and/or pontocerebellar hypoplasia to identify the known pathogenic variants according to the ClinVar database (Supplementary Table S4). There were no more known pathogenic or likely pathogenic variants in the disease-causing genes other than the *CASK* gene (Supplementary Table S4).

## Discussion

In the present study, we identified a *CASK* missense variant (c.1882G>C, p.Asp628His) in a Chinese pedigree by whole-exome sequencing and Sanger verification. Bioinformatics analysis showed codon 628 was located within a highly conserved region in various species, and the structure model showed this variant could disturb the SH3 domain and destroy the stability of CASK. This variant is neither reported in literature nor registered in HGMD, indicating a novel one. Our findings expand the variant spectrum of *CASK* in MICPCH patients.

The CASK is a multi-domain scaffolding protein that belongs to the membrane-associated guanylate kinase (MAGUK) family. It contains an N-terminal calcium/calmodulin-dependent kinase (CaMK) domain, followed by two L27 domains, a PDZ motif, an SH3 domain, and a guanylate kinase domain (LaConte et al., 2019). Variants in the *CASK* gene are associated with many neurodevelopmental disorders. *CASK* loss-of-function variants are often associated with MICPCH, which are often found in females and are considered to be lethal in males. Hypomorphic *CASK* missense variants are often associated with X-linked intellectual disability (XLID) with or without nystagmus, which can be found in both males and females. For males, who only have a single allele of the *CASK* gene, the clinical presentations of patients who carried the *CASK* missense variants are often more severe than those of females, which are variable from mild XLID to MICPCH (Takanashi et al., 2012; Moog et al., 2015; Hayashi et al., 2017).

The previously reported *CASK* missense variants in male patients are summarized in Supplementary Table S1 (Tarpey et al., 2009; Hackett et al., 2010; Saitsu et al., 2012; Takanashi et al., 2012; de Kovel et al., 2016; Reinstein et al., 2016; Deciphering Developmental Disorders Study, 2017; Hayashi et al., 2017; Seto et al., 2017; Hauer et al., 2018; Carraro et al., 2019; Devi et al., 2019; Kerr et al., 2019; Long et al., 2019; Ibarluzea et al., 2020; Islam et al., 2020; Rochtus et al., 2020; Pan et al., 2021). The missense variants seem to occur randomly in the supradomains, including CaK, PDZ, SH3, and GuK domains, indicating that the genotype-phenotype relationship in MICPCH is still unclear. Previous research established that the MICPCH phenotype is caused by the breakdown of the CASK-neurexin connection (LaConte et al., 2018). The CASK-neurexin interaction was traditionally suggested to be mediated by the binding of a few residues at the end of neurexin’s cytoplasmic tail to CASK’s PDZ domain (LaConte et al., 2016; LaConte et al., 2018), and missense variants located on the PDZ domain may be linked to developmental disorders with/without microcephaly (Seto et al., 2017). However, other studies show that additional domains, such as the SH3-Guk domain, are necessary for the interaction (Li et al., 2014; Reissner and Missler, 2014). A PDZ-mediated connection can be broken by variants in the SH3 domain, which can also cause protein aggregation (LaConte et al., 2018; Kerr et al., 2019). In our pedigree, the p.D628H variant was found in the highly conserved CASK SH3 domain. Because histidine is a non-polar amino acid and bigger than the negatively charged aspartic acid, protein structure modeling indicated that this amino acid replacement would result in a loss in the stability of the CASK protein. This missense variant likely produces secondary and tertiary structural defects and disrupts CASK-neurexin interaction, leading to the MICPCH phenotype.

Many complex genetic and environmental factors can ultimately contribute to the incidence of intellectual disability and dystaxia, making it essential that the specific etiological basis for these outcomes in individual patients be established. Accordingly, we initially conducted SNP array and capillary electrophoresis to exclude the pathogenic CNVs and expansion of CAG repeats in the *SCA* gene. With no positive results, subsequent whole-exome sequencing was performed, identifying a likely pathogenic missense variant in the *CASK* gene. Thus, for pediatric patients with one or more congenital anomalies, developmental delay, or intellectual disability, the whole exome/whole genome sequencing should be considered as a first- or second-tier test instead of chromosomal microarray (ManickamKMcClain et al., 2021).

The inheritance pattern of CASK-related disorder is X-linked. In males, X-linked pathogenic variants affect every cell, whereas, in females, they often affect half of the cells due to random X inactivation. Thus, the phenotype of the patients carrying pathogenic variants of CASK is usually more severe in males than females (Hackett et al., 2010), and the phenotype severity in females usually depends on the proportion of CASK-deficient cells (Moog et al., 2011; Seto et al., 2017). In our family, two male patients exhibited MICPCH, whereas two female patients exhibited mild intellectual disability. The limitation of the present study is that, due to the lack of additional blood samples from two female family members, we were unable to conduct X inactivation analyses. We speculated that two female patients have a skewed X-inactivation pattern favoring the expression of the mutated allele, resulting in a mild phenotype.

In summary, we have identified a novel p.Asp628His variant of the *CASK* gene in a Chinese family. The variant identified in this study was a missense variant located on the SH3 domain in male patients associated with MICPCH manifestations. Our finding expands the known *CASK* variant spectrum while additionally demonstrating the ease, rapidity, and accuracy of using targeted next-generation sequencing assays to facilitate the clinical diagnosis of patients affected by heritable diseases.

## Data Availability

The datasets for this article are not publicly available due to concerns regarding participant/patient anonymity. The datasets should be available after proper request to the corresponding author.
