# Supplementary material for: Case Report: Identification of a novel CASK missense variant in a Chinese family with MICPCH
Source: Front Genet. 2022 Aug 25;13:933785. doi: 10.3389/fgene.2022.933785 (PMC9452731; doi:10.3389/fgene.2022.933785)
Supplement: Supplementary file 1 [file Table4.DOC]

Supplemental Table 4 The 78 genes that might cause microcephaly and/or pontocerebellar hypoplasia

| **Gene** | **Associated phenotypes** | **Inheritance** |
| --- | --- | --- |
| AKT3 | Megalencephaly-polymicrogyria-polydactyly-hydrocephalus syndrome | AD |
| AMPD2 | Pontocerebellar hypoplasia type 9, Spastic paraplegia 63 | AR |
| ASPM | Microcephaly | AR |
| ASXL1 | Bohring-Opitz syndrome | AD |
| ASXL3 | Bainbridge-Ropers syndrome | AD |
| ATR | Cutaneous telangiectasia and cancer syndrome, Seckel syndrome | AD/AR |
| CASK | Mental retardation and microcephaly with pontine and cerebellar hypoplasia, FG syndrome, Mental retardation | XL |
| CCDC47 | Microcephaly, Malformations | AR |
| CDK5RAP2 | Microcephaly | AR |
| CENPF | Ciliary dyskinesia -Lethal Ciliopathy | AR |
| CENPJ | Seckel syndrome, Microcephaly | AR |
| CEP152 | Seckel syndrome, Microcephaly | AR |
| CEP63 | Seckel syndrome | AR |
| CSNK2A1 |  | AD |
| DONSON | Microcephaly, short stature, and limb abnormalities (MISSLA), Microcephaly-Micromelia syndrome | AR |
| DYNC1H1 | Spinal muscular atrophy, Charcot-Marie-Tooth disease, Mental retardation | AD |
| DYRK1A | Mental retardation | AD |
| EFTUD2 | Mandibulofacial dysostosis with microcephaly, Esophageal atresia, syndromic | AD |
| EXOSC3 | Pontocerebellar hypoplasia | AR |
| GFM1 | Combined oxidative phosphorylation deficiency | AR |
| GPT2 | Mental retardation, autosomal recessive 49, Microcephaly, Spastic paraplegia | AR |
| [KANSL1](https://blueprintgenetics.com/pseudogene/#KANSL1)* | Koolen-de Vries syndrome | AD |
| KATNB1 | Lissencephaly 6, with microcephaly | AR |
| KIF11 | Microcephaly | AD |
| LIG4 | Severe combined immunodeficiency with sensitivity to ionizing radiation, LIG4 syndrome | AR |
| MBD5 | Mental retardation | AD |
| [MCPH1](https://blueprintgenetics.com/pseudogene/#MCPH1)# | Microcephaly | AR |
| MED17 | Microcephaly, postnatal progressive, with seizures and brain atrophy | AR |
| MFSD2A | Microcephaly 15, primary, autosomal recessive | AR |
| [MIPEP](https://blueprintgenetics.com/pseudogene/#MIPEP)* | Combined oxidative phosphorylation deficiency 31 | AR |
| MRE11A | Ataxia-telangiectasia-like disorder-1 | AR |
| MYCN | Feingold syndrome | AD |
| MYO18B | Klippel-Feil syndrome 4, autosomal recessive, with myopathy and facial dysmorphism | AR |
| NCAPD3 | Microcephaly | AR |
| NDE1 | Microhydranencephaly, Lissencephaly | AR |
| NHEJ1 | Severe combined immunodeficiency with microcephaly, growth retardation, and sensitivity to ionizing radiation | AR |
| OPHN1 | Mental retardation, with cerebellar hypoplasia and distinctive facial appearance | XL |
| PAFAH1B1 | Lissencephaly, Subcortical laminar heterotopia | AD |
| PCDH12 | Microcephaly | AR |
| PCLO | Pontocerebellar hypoplasia | AR |
| PCNT | Microcephalic osteodysplastic primordial dwarfism | AR |
| PHGDH | Neu-Laxova syndrome 1 | AR |
| PLK4 | Microcephaly and chorioretinopathy, autosomal recessive 2 | AR |
| PNKP | Epileptic encephalopathy, early infantile, Ataxia-oculomotor | AR |
| POMT1 | Muscular dystrophy-dystroglycanopathy | AR |
| PQBP1 | Renpenning syndrome | XL |
| QARS | Microcephaly, progressive, seizures, and cerebral and cerebellar atrophy | AR |
| RARS2 | Pontocerebellar hypoplasia | AR |
| RTTN | Microcephaly, short stature, and polymicrogyria with or without seizures | AR |
| SEPSECS | Pontocerebellar hypoplasia, type 2D | AR |
| SLC1A4 | Spastic tetraplegia, thin corpus callosum, and progressive microcephaly | AR |
| SMARCA2 | Nicolaides-Baraitser syndrome | AD |
| SMARCE1 | Coffin-Siris syndrome | AD |
| SOX11 | Mental retardation, autosomal dominant 27 | AD |
| STAG2 | Congenital heart defects, dysmorphic facial features, and intellectual developmental disorder | XL |
| STAMBP | Microcephaly-capillary malformation syndrome | AR |
| STIL | Microcephaly | AR |
| TBC1D20 | Warburg micro syndrome 4 | AR |
| TBC1D23 | Pontocerebellar hypoplasia, type 11 |  |
| THOC6 | Microcephaly | AR |
| TMTC3 | Lissencephaly 8 |  |
| TOE1 | Pontocerebellar hypoplasia type 7 |  |
| TOP3A |  |  |
| TRMT10A | Microcephaly, short stature, and impaired glucose metabolism 1 | AR |
| [TSEN2](https://blueprintgenetics.com/pseudogene/#TSEN2)# | Pontocerebellar hypoplasia | AR |
| TSEN54 | Pontocerebellar hypoplasia | AR |
| [TUBB](https://blueprintgenetics.com/pseudogene/#TUBB)* | Congenital symmetric circumferential skin creases 1, Cortical dysplasia, complex, with other brain malformations 6 | AD |
| [TUBB2B](https://blueprintgenetics.com/pseudogene/#TUBB2B)#* | Polymicrogyria, asymmetric | AD |
| TUBGCP4 | Microcephaly and chorioretinopathy, autosomal recessive 3 | AR |
| TUBGCP6 | Microcephaly and chorioretinopathy, autosomal recessive 1 | AR |
| UBE3B | Blepharophimosis-Ptosis-Intellectual-Disability syndrome (Kaufman oculocerebrofacial syndrome) | AR |
| VARS | Early-onset progressive encephalopathy with brain atrophy and thin corpus callosum (PEBAT), Encephalopathy, progressive | AR |
| VRK1 | Pontocerebellar hypoplasia | AR |
| WDR62 | Microcephaly | AR |
| WDR73 | Galloway-Mowat syndrome | AR |
| XRCC4 | Short stature, microcephaly, and endocrine dysfunction | AR |
| ZNF148 | Global developmental delay, absent or hypoplastic corpus callosum, and dysmorphic facies (GDACCF) |  |
| ZNF335 | Microcephaly 10, primary, autosomal recessive | AR |

**References：**

1. Namavar, Y., Barth, PG., Poll-The, BT., Baas, F. (2011). Classification, diagnosis and potential mechanisms in Pontocerebellar Hypoplasia. Orphanet J Rare Dis. 6(1), 50. doi:10.1186/1750-1172-6-50

2. Siskos, N., Stylianopoulou, E., Skavdis, G., Grigoriou, ME. (2021). Molecular Genetics of Microcephaly Primary Hereditary: An Overview. Brain Sci. 11(5): 581. doi:10.3390/brainsci11050581

3. Faheem, M., Naseer, M. I., Rasool, M., Chaudhary, A. G., Kumosani, T. A., Ilyas, A. M., et al. (2015). Molecular genetics of human primary microcephaly: an overview. BMC Medical Genomics, 8(S1). doi:10.1186/1755-8794-8-s1-s4
