# Supplementary material for: Case Report: Identification of a novel CASK missense variant in a Chinese family with MICPCH
Source: Front Genet. 2022 Aug 25;13:933785. doi: 10.3389/fgene.2022.933785 (PMC9452731; doi:10.3389/fgene.2022.933785)
Supplement: Supplementary file 2 [file Table1.DOCX]

Supplemental table 1 Clinical features of the patients in pedigree

| ID | Gender | Age | Dystaxia | Intellectual isability | Nystagmus | Biparietal diameter |
| --- | --- | --- | --- | --- | --- | --- |
| I2 | F | 71 | - | - | - |  |
| I3 | F | 67 | - | + | - |  |
| Ⅱ1 | M | 45 | - | - | - |  |
| Ⅱ2 | M | 42 | - | - | + |  |
| Ⅱ3 | M | 39 | - | - | + |  |
| Ⅱ4 | F | 27 | - | + | - |  |
| Ⅲ1 | M | 8 | + | + | - | 12.5 ^a^ |
| Ⅲ2 | M | 3 | + | + | - | 11 ^b^ |

M, male; F, female; +, present; -, absent.

^a^ Less than normal value (14 cm); ^b^ less than normal value (13.3 cm).
