# Supplementary material for: Case Report: Identification of a novel CASK missense variant in a Chinese family with MICPCH
Source: Front Genet. 2022 Aug 25;13:933785. doi: 10.3389/fgene.2022.933785 (PMC9452731; doi:10.3389/fgene.2022.933785)
Supplement: Supplementary file 3 [file Table3.DOC]

Supplemental Table 3 The number of variants after each filtering step

| Step | Variants |
| --- | --- |
| Initial | 71,106 |
| 1. exclude variants outside exonic and splicing regions | 25,655 |
| 2. exclude variants with MAF* > 0.01 | 1,523 |
| 3. exclude synonymous variants | 1,010 |
| 4.include the variants in inherited in X-linked pattern# | 8 |

*MAF:Minor Allele Frequency in 1000 genome and Exome Aggregation Consortium database
