# Supplementary figures and images for: Case Report: Identification of a novel CASK missense variant in a Chinese family with MICPCH
Source: Front Genet. 2022 Aug 25;13:933785. doi: 10.3389/fgene.2022.933785 (PMC9452731; doi:10.3389/fgene.2022.933785)

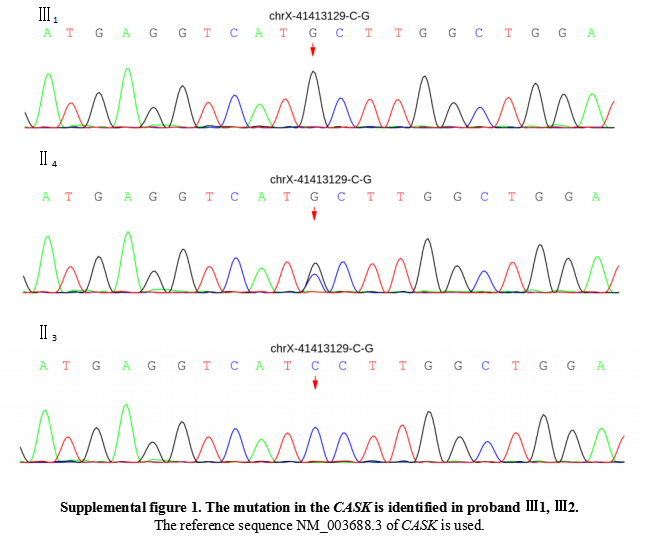

Supplement: Supplementary file 4 [file Image1.TIF]
