# Supplementary material for: Case Report: Identification of a novel CASK missense variant in a Chinese family with MICPCH
Source: Front Genet. 2022 Aug 25;13:933785. doi: 10.3389/fgene.2022.933785 (PMC9452731; doi:10.3389/fgene.2022.933785)
Supplement: Supplementary file 5 [file Table2.DOCX]

Supplemental table 2 CAG repeat expansion of SCA in proband III1

| Type | Repeat number | Normal reference range |
| --- | --- | --- |
| SCA1 | 26, 26 | <39 |
| SCA2 | 20, 20 | <33 |
| SCA3 | 7, 21 | <44 |
