# Supplementary material for: Case Report: Identification of a novel CASK missense variant in a Chinese family with MICPCH
Source: Front Genet. 2022 Aug 25;13:933785. doi: 10.3389/fgene.2022.933785 (PMC9452731; doi:10.3389/fgene.2022.933785)
Supplement: Supplementary file 6 [file DataSheet1.PDF]

```
                           1         10        20        30        40        50
Macaca_mulatta             .ADDDVLFEDVYELCEVIGKGPFSVVRRCINRETGQQFAVKIVDVAKFTSSP....GLST
Macaca_fascicularis        MADDDVLFEDVYELCEVIGKGPFSVVRRCINRETGQQFAVKIVDVAKFTSSP....GLST
Echinops_telfairi          MADDDVLFEDVYELCEVIGKGPFSVVRRCINRETGQQFAVKIVDVAKFTSSP....GLST
Ornithorhynchus_anatinus   MADDDVLFEDVYELCEVIGKGPFSVVRRCINRETGQQFAVKIVDVAKFTSSP....GLST
Homo_sapiens               MADDDVLFEDVYELCEVIGKGPFSVVRRCINRETGQQFAVKIVDVAKFTSSP....GLST
Cavia_porcellus            MADDDVLFEDVYELCEVIGKGPFSVVRRCINRETGQQFAVKIVDVAKFTSSP....GLST
Suricata_suricatta         MADDDVLFEDVYELCEVIGKGPFSVVRRCINRETGQQFAVKIVDVAKFTSSP....GLST
Macaca_nemestrina          MADDDVLFEDVYELCEVIGKGPFSVVRRCINRETGQQFAVKIVDVAKFTSSP....GLST
Tupaia_chinensis           MADDDVLFEDVYELCEVIGKGPFSVVRRCINRETGQQFAVKIVDVAKFTSSP....GLST
Bos_mutus                  MADDDVLFEDVYELCEVIGKGPFSVVRRCINRETGQQFAVKIVDVAKFTSSP....GLST
Pipistrellus_kuhlii        MADDDVLFEDVYELCEVIGKGPFSVVRRCINRETGQQFAVKIVDVAKFTSSP....GLST
Capra_hircus               MADDDVLFEDVYELCEVIGKGPFSVVRRCINRETGQQFAVKIVDVAKFTSSP....GLST
Ovis_aries                 MADDDVLFEDVYELCEVIGKGPFSVVRRCINRETGQQFAVKIVDVAKFTSSP....GLST
Equus_caballus             MADDDVLFEDVYELCKVIGKGPFSVVRRCINRETGQQFAVKIVDVAKFTSSP....GLST
Balaenoptera_musculus      MADDDVLFEDVYELCEVIGKGPFSVVRRCINRETGQQFAVKIVDVAKFTSSP....GLST
Rattus_norvegicus          MADDDVLFEDVYELCEVIGKGPFSVVRRCINRETGQQFAVKIVDVAKFTSSPGLST....
Microtus_oregoni           MADDDVLFEDVYELCEVIGKGPFSVVRRCINRETGQQFAVKIVDVAKFTSSPGLST....
Microtus_ochrogaster       MADDDVLFEDVYELCEVIGKGPFSVVRRCINRETGQQFAVKIVDVAKFTSSPGLST....
Pongo_abelii               MADDDVLFEDVYELCEVIGKGPFSVVRRCINRETGQQFAVKIVDVAKFTSSPG.......
Zalophus_californianus     MADDDVLFEDVYELCEVIGKGPFSVVRRCINRETGQQFAVKIVDVAKFTSSPG.......
Delphinapterus_leucas      MADDDVLFEDVYELCEVIGKGPFSVVRRCINRETGQQFAVKIVDVAKFTSSPG.......
Mus_musculus               MADDDVLFEDVYELCEVIGKGPFSVVRRCINRETGQQFAVKIVDVAKFTSSPG.......

                                   60         70        80        90        100
Macaca_mulatta             ......EDLKREASICHMLKHPHIVELLETYSSDGMLYMVFEFMDGADLCFEIVKRADAG
Macaca_fascicularis        ......EDLKREASICHMLKHPHIVELLETYSSDGMLYMVFEFMDGADLCFEIVKRADAG
Echinops_telfairi          ......EDLKREASICHMLKHPHIVELLETYSSDGMLYMVFEFMDGADLCFEIVKRADAG
Ornithorhynchus_anatinus   ......EDLKREASICHMLKHPHIVELLETYSSDGMLYMVFEFMDGADLCFEIVKRADAG
Homo_sapiens               ......EDLKREASICHMLKHPHIVELLETYSSDGMLYMVFEFMDGADLCFEIVKRADAG
Cavia_porcellus            ......EDLKREASICHMLKHPHIVELLETYSSDGMLYMVFEFMDGADLCFEIVKRADAG
Suricata_suricatta         ......EDLKREASICHMLKHPHIVELLETYSSDGMLYMVFEFMDGADLCFEIVKRADAG
Macaca_nemestrina          ......EDLKREASICHMLKHPHIVELLETYSSDGMLYMVFEFMDGADLCFEIVKRADAG
Tupaia_chinensis           ......EDLKREASICHMLKHPHIVELLETYSSDGMLYMVFEFLDGADLCFEIVKRADAG
Bos_mutus                  ......EDLKREASICHMLKHPHIVELLETYSSDGMLYMVFEFMDGADLCFEIVKRADAG
Pipistrellus_kuhlii        ......EDLKREASICHMLKHPHIVELLETYSSDGMLYMVFEFMDGADLCFEIVKRADAG
Capra_hircus               ......EDLKREASICHMLKHPHIVELLETYSSDGMLYMVFEFMDGADLCFEIVKRADAG
Ovis_aries                 EGKRWISNLKREASICHMLKHPHIVELLETYSSDGMLYMVFEFMDGADLCFEIVKRADAG
Equus_caballus             EGKRWISNLKREASICHMLKHPHIVELLETYSSDGMLYMVFEFMDGADLCFEIVKRADAG
Balaenoptera_musculus      EGKRWISNLKREASICHMLKHPHIVELLETYSSDGMLYMVFEFMDGADLCFEIVKRADAG
Rattus_norvegicus          ......EDLKREASICHMLKHPHIVELLETYSSDGMLYMVFEFMDGADLCFEIVKRADAG
Microtus_oregoni           ......EDLKREASICHMLKHPHIVELLETYSSDGMLYMVFEFMDGADLCFEIVKRADAG
Microtus_ochrogaster       ......EDLKREASICHMLKHPHIVELLETYSSDGMLYMVFEFMDGADLCFEIVKRADAG
Pongo_abelii               ...LSTEDLKREASICHMLKHPHIVELLETYSSDGMLYMVFEFMDGADLCFEIVKRADAG
Zalophus_californianus     ...LSTEDLKREASICHMLKHPHIVELLETYSSDGMLYMVFEFMDGADLCFEIVKRADAG
Delphinapterus_leucas      ...LSTEDLKREASICHMLKHPHIVELLETYSSDGMLYMVFEFMDGADLCFEIVKRADAG
Mus_musculus               ...LSTEDLKREASICHMLKHPHIVELLETYSSDGMLYMVFEFMDGADLCFEIVKRADAG

                         110       120       130       140       150       160
Macaca_mulatta             FVYSEAVASHYMRQILEALRYCHDNNIIHRDVKPHCVLLASKENSAPVKLGGFGVAIQLG
Macaca_fascicularis        FVYSEAVASHYMRQILEALRYCHDNNIIHRDVKPHCVLLASKENSAPVKLGGFGVAIQLG
Echinops_telfairi          FVYSEAVASHYMRQILEALRYCHDNNIIHRDVKPHCVLLASKENSAPVKLGGFGVAIQLG
Ornithorhynchus_anatinus   FVYSEAVASHYMRQILEALRYCHDNNIIHRDVKPHCVLLASKENSAPVKLGGFGVAIQLG
Homo_sapiens               FVYSEAVASHYMRQILEALRYCHDNNIIHRDVKPHCVLLASKENSAPVKLGGFGVAIQLG
Cavia_porcellus            FVYSEAVASHYMRQILEALRYCHDNNIIHRDVKPHCVLLASKENSAPVKLGGFGVAIQLG
Suricata_suricatta         FVYSEAVASHYMRQILEALRYCHDNNIIHRDVKPHCVLLASKENSAPVKLGGFGVAIQLG
Macaca_nemestrina          FVYSEAVASHYMRQILEALRYCHDNNIIHRDVKPHCVLLASKENSAPVKLGGFGVAIQLG
Tupaia_chinensis           FVYSEAVASHYMRQILEALRYCHDNNIIHRDVKPHCVLLASKENSAPVKLGGFGVAIQLG
Bos_mutus                  FVYSEAVASHYMRQILEALRYCHDNNIIHRDVKPHCVLLASKENSAPVKLGGFGVAIQLG
Pipistrellus_kuhlii        FVYSEAVASHYMRQILEALRYCHDNNIIHRDVKPHCVLLASKENSAPVKLGGFGVAIQLG
Capra_hircus               FVYSEAVASHYMRQILEALRYCHDNNIIHRDVKPHCVLLASKENSAPVKLGGFGVAIQLG
Ovis_aries                 FVYSEAVASHYMRQILEALRYCHDNNIIHRDVKPHCVLLASKENSAPVKLGGFGVAIQLG
Equus_caballus             FVYSEAVASHYMRQILEALRYCHDNNIIHRDVKPHCVLLASKENSAPVKLGGFGVAIQLG
Balaenoptera_musculus      FVYSEAVASHYMRQILEALRYCHDNNIIHRDVKPHCVLLASKENSAPVKLGGFGVAIQLG
Rattus_norvegicus          FVYSEAVASHYMRQILEALRYCHDNNIIHRDVKPHCVLLASKENSAPVKLGGFGVAIQLG
Microtus_oregoni           FVYSEAVASHYMRQILEALRYCHDNNIIHRDVKPHCVLLASKENSAPVKLGGFGVAIQLG
Microtus_ochrogaster       FVYSEAVASHYMRQILEALRYCHDNNIIHRDVKPHCVLLASKENSAPVKLGGFGVAIQLG
Pongo_abelii               FVYSEAVASHYMRQILEALRYCHDNNIIHRDVKPHCVLLASKENSAPVKLGGFGVAIQLG
Zalophus_californianus     FVYSEAVASHYMRQILEALRYCHDNNIIHRDVKPHCVLLASKENSAPVKLGGFGVAIQLG
Delphinapterus_leucas      FVYSEAVASHYMRQILEALRYCHDNNIIHRDVKPHCVLLASKENSAPVKLGGFGVAIQLG
Mus_musculus               FVYSEAVASHYMRQILEALRYCHDNNIIHRDVKPHCVLLASKENSAPVKLGGFGVAIQLG
```

```
                          170       180       190       200       210       220
Macaca_mulatta            ESGLVAGGRVGTPHFMAPEVVKREPYGKPVDVWGCGVILFILLSGCLPFYGTKERLFEGI
Macaca_fascicularis       ESGLVAGGRVGTPHFMAPEVVKREPYGKPVDVWGCGVILFILLSGCLPFYGTKERLFEGI
Echinops_telfairi         ESGLVAGGRVGTPHFMAPEVVKREPYGKPVDVWGCGVILFILLSGCLPFYGTKERLFEGI
Ornithorhynchus_anatinus  ESGLVAGGRVGTPHFMAPEVVKREPYGKPVDVWGCGVILFILLSGCLPFYGTKERLFEGI
Homo_sapiens              ESGLVAGGRVGTPHFMAPEVVKREPYGKPVDVWGCGVILFILLSGCLPFYGTKERLFEGI
Cavia_porcellus           ESGLVAGGRVGTPHFMAPEVVKREPYGKPVDVWGCGVILFILLSGCLPFYGTKERLFEGI
Suricata_suricatta        ESGLVAGGRVGTPHFMAPEVVKREPYGKPVDVWGCGVILFILLSGCLPFYGTKERLFEGI
Macaca_nemestrina         ESGLVAGGRVGTPHFMAPEVVKREPYGKPVDVWGCGVILFILLSGCLPFYGTKERLFEGI
Tupaia_chinensis          ESGLVAGGRVGTPHFMAPEVVKREPYGKPVDVWGCGVILFILLSGCLPFYGTKERLFEGI
Bos_mutus                 ESGLVAGGRVGTPHFMAPEVVKREPYGKPVDVWGCGVILFILLSGCLPFYGTKERLFEGI
Pipistrellus_kuhlii       ESGLVAGGRVGTPHFMAPEVVKREPYGKPVDVWGCGVILFILLSGCLPFYGTKERLFEGI
Capra_hircus              ESGLVAGGRVGTPHFMAPEVVKREPYGKPVDVWGCGVILFILLSGCLPFYGTKERLFEGI
Ovis_aries                ESGLVAGGRVGTPHFMAPEVVKREPYGKPVDVWGCGVILFILLSGCLPFYGTKERLFEGI
Equus_caballus            ESGLVAGGRVGTPHFMAPEVVKREPYGKPVDVWGCGVILFILLSGCLPFYGTKERLFEGI
Balaenoptera_musculus     ESGLVAGGRVGTPHFMAPEVVKREPYGKPVDVWGCGVILFILLSGCLPFYGTKERLFEGI
Rattus_norvegicus         ESGLVAGGRVGTPHFMAPEVVKREPYGKPVDVWGCGVILFILLSGCLPFYGTKERLFEGI
Microtus_oregoni          ESGLVAGGRVGTPHFMAPEVVKREPYGKPVDVWGCGVILFILLSGCLPFYGTKERLFEGI
Microtus_ochrogaster      ESGLVAGGRVGTPHFMAPEVVKREPYGKPVDVWGCGVILFILLSGCLPFYGTKERLFEGI
Pongo_abelii              ESGLVAGGRVGTPHFMAPEVVKREPYGKPVDVWGCGVILFILLSGCLPFYGTKERLFEGI
Zalophus_californianus    ESGLVAGGRVGTPHFMAPEVVKREPYGKPVDVWGCGVILFILLSGCLPFYGTKERLFEGI
Delphinapterus_leucas     ESGLVAGGRVGTPHFMAPEVVKREPYGKPVDVWGCGVILFILLSGCLPFYGTKERLFEGI
Mus_musculus              ESGLVAGGRVGTPHFMAPEVVKREPYGKPVDVWGCGVILFILLSGCLPFYGTKERLFEGI
```

```
                          230       240       250       260       270       280
Macaca_mulatta            IKGKYKMNPRQWSHISESAKDLVRRMLMLDPAERITVYEALNHPWLKERDRYAYKIHLPE
Macaca_fascicularis       IKGKYKMNPRQWSHISESAKDLVRRMLMLDPAERITVYEALNHPWLKERDRYAYKIHLPE
Echinops_telfairi         IKGKYKLNPRQWSHISESAKDLVRRMLMLDPAERITVYEALNHPWLKERDRYAYKIHLPE
Ornithorhynchus_anatinus  IKGKYKMNPRQWSHISESAKDLVRRMLMLDPAERITVYEALNHPWLKERDRYAYKIHLPE
Homo_sapiens              IKGKYKMNPRQWSHISESAKDLVRRMLMLDPAERITVYEALNHPWLKERDRYAYKIHLPE
Cavia_porcellus           IKGKYKMNPRQWSHISESAKDLVRRMLMLDPAERITVYEALNHPWLKERDRYAYKIHLPE
Suricata_suricatta        IKGKYKMNPRQWSHISESAKDLVRRMLMLDPAERITVYEALNHPWLKERDRYAYKIHLPE
Macaca_nemestrina         IKGKYKMNPRQWNHISESAKDLVRRMLMLDPAERITVYEALNHPWLKERDRYAYKIHLPE
Tupaia_chinensis          IKGKYKMKPWQWSHISESAKDLVRRMLMLDPAERITVYEALNHPWLKERDRYAYKIHLPE
Bos_mutus                 IKGKYKMNPRQWSHISESAKDLVRRMLMLDPAERITVYEALNHPWLKERDRYAYKIHLPE
Pipistrellus_kuhlii       IKGKYKMNPRQWSHISESAKDLVRRMLMLDPAERITVYEALNHPWLKERDRYAYKIHLPE
Capra_hircus              IKGKYKMNPRQWSHISESAKDLVRRMLMLDPAERITVYEALNHPWLKERDRYAYKIHLPE
Ovis_aries                IKGKYKMNPRQWSHISESAKDLVRRMLMLDPAERITVYEALNHPWLKERDRYAYKIHLPE
Equus_caballus            IKGKYKMNPRQWSHISESAKDLVRRMLMLDPAERITVYEALNHPWLKERDRYAYKIHLPE
Balaenoptera_musculus     IKGKYKMNPRQWSHISESAKDLVRRMLMLDPAERITVYEALNHPWLKERDRYAYKIHLPE
Rattus_norvegicus         IKGKYKMNPRQWSHISESAKDLVRRMLMLDPAERITVYEALNHPWLKERDRYAYKIHLPE
Microtus_oregoni          IKGKYKMNPRQWSHISESAKDLVRRMLMLDPAERITVYEALNHPWLKERDRYAYKIHLPE
Microtus_ochrogaster      IKGKYKMNPRQWSHISESAKDLVRRMLMLDPAERITVYEALNHPWLKERDRYAYKIHLPE
Pongo_abelii              IKGKYKMNPRQWSHISESAKDLVRRMLMLDPAERITVYEALNHPWLKERDRYAYKIHLPE
Zalophus_californianus    IKGKYKMNPRQWSHISESAKDLVRRMLMLDPAERITVYEALNHPWLKERDRYAYKIHLPE
Delphinapterus_leucas     IKGKYKMNPRQWSHISESAKDLVRRMLMLDPAERITVYEALNHPWLKERDRYAYKIHLPE
Mus_musculus              IKGKYKMNPRQWSHISESAKDLVRRMLMLDPAERITVYEALNHPWLKERDRYAYKIHLPE
```

```
                          290       300       310       320       330       340
Macaca_mulatta            TVEQLRKFNARRKLKGAVLAAVSSHKFNSFYGDPPEELPDFSEDPTSSGSLSATKSFSQV
Macaca_fascicularis       TVEQLRKFNARRKLKGAVLAAVSSHKFNSFYGDPPEELPDFSEDPTSSGSLSATKSFSQV
Echinops_telfairi         TVEQLRKFNARRKLKGAVLAAVSSHKFNSFYGDPPEELADFSEDPTSSGLLAAERAVSQV
Ornithorhynchus_anatinus  TVEQLRKFNARRKLKGAVLAAVSSHKFNSFYGDPPEELPDFSEDPTSSGLLTAERAVSQV
Homo_sapiens              TVEQLRKFNARRKLKGAVLAAVSSHKFNSFYGDPPEELPDFSEDPTSSGLLAAERAVSQV
Cavia_porcellus           TVEQLRKFNARRKLKGAVLAAVSSHKFNSFYGDPPEELPDFSEDPTSSGLLAAERAVSQV
Suricata_suricatta        TVEQLRKFNARRKLKGAVLAAVSSHKFNSFYGDPPEELPDFSEDPTSSGLLAAERAVSQV
Macaca_nemestrina         TVEQLRKFNARRKLKGAVLAAVSSHKFNSFYGDPPEELPDFSEDPTSSGLLAAERAVSQV
Tupaia_chinensis          TVEQLRKFNARRKLKGAVLAAVSSHKFNSFYGDPPEELPDFSEDPTSSGLLAAERAVSQV
Bos_mutus                 TVEQLRKFNARRKLKGAVLAAVSSHKFNSFYGDPPEELPDFSEDPTSSGLLAAERAVSQV
Pipistrellus_kuhlii       TVEQLRKFNARRKLKGAVLAAVSSHKFNSFYGDPPEELPDFSEDPTSSGLLAAERAVSQV
Capra_hircus              TVEQLRKFNARRKLKGAVLAAVSSHKFNSFYGDPPEELPDFSEDPTSSGLLAAERAVSQV
Ovis_aries                TVEQLRKFNARRKLKGAVLAAVSSHKFNSFYGDPPEELPDFSEDPTSSGLLAAERAVSQV
Equus_caballus            TVEQLRKFNARRKLKGAVLAAVSSHKFNSFYGDPPEELPDFSEDPTSSGLLAAERAVSQV
Balaenoptera_musculus     TVEQLRKFNARRKLKGAVLAAVSSHKFNSFYGDPPEELPDFSEDPTSSGLLAAERAVSQV
Rattus_norvegicus         TVEQLRKFNARRKLKGAVLAAVSSHKFNSFYGDPPEELPDFSEDPTSSGLLAAERAVSQV
Microtus_oregoni          TVEQLRKFNARRKLKGAVLAAVSSHKFNSFYGDPPEELPDFSEDPTSSGLLAAERAVSQV
Microtus_ochrogaster      TVEQLRKFNARRKLKGAVLAAVSSHKFNSFYGDPPEELPDFSEDPTSSGLLAAERAVSQV
Pongo_abelii              TVEQLRKFNARRKLKGAVLAAVSSHKFNSFYGDPPEELPDFSEDPTSSGLLAAERAVSQV
Zalophus_californianus    TVEQLRKFNARRKLKGAVLAAVSSHKFNSFYGDPPEELPDFSEDPTSSGLLAAERAVSQV
Delphinapterus_leucas     TVEQLRKFNARRKLKGAVLAAVSSHKFNSFYGDPPEELPDFSEDPTSSGLLAAERAVSQV
Mus_musculus              TVEQLRKFNARRKLKGAVLAAVSSHKFNSFYGDPPEELPDFSEDPTSSGLLAAERAVSQV
```

```
                          350       360       370       380       390       400
Macaca_mulatta            LDSLEEIHALTDCSEKDLDFLHSVFQDQHLHTLLDLYDKINTKSSPQIRNPPSDAVQRAK
Macaca_fascicularis       LDSLEEIHALTDCSEKDLDFLHSVFQDQHLHTLLDLYDKINTKSSPQIRNPPSDAVQRAK
Echinops_telfairi         LDSLEEIHALTDCSEKDLDFLHSVFQDQHLHTLLDLYDKINTKSSPQIRNPPSDAVQRAK
Ornithorhynchus_anatinus  LDSLEEIHALTDCSEKDLDFLHSVFQDQHLHTLLDLYDKINTKSSPQIRNPPSDAVQRAK
Homo_sapiens              LDSLEEIHALTDCSEKDLDFLHSVFQDQHLHTLLDLYDKINTKSSPQIRNPPSDAVQRAK
Cavia_porcellus           LDSLEEIHALTDCSEKDLDFLHSVFQDQHLHTLLDLYDKINTKSSPQIRNPPSDAVQRAK
Suricata_suricatta        LDSLEEIHALTDCSEKDLDFLHSVFQDQHLHTLLDLYDKINTKSSPQIRNPPSDAVHRAK
Macaca_nemestrina         LDSLEEIHALTDCSEKDLDFLHSVFQDQHLHTLLDLYDKINTKSSPQIRNPPSDAVQRAK
Tupaia_chinensis          LDSLEEIHALTDCSEKDLDFLHSVFQDQHLHTLLDLYDKINTKSSPQIRNPPSDAVQRAK
Bos_mutus                 LDSLEEIHALTDCSEKDLDFLHSVFQDQHLHTLLDLYDKINTKSSPQIRNPPSDAVQRAK
Pipistrellus_kuhlii       LDSLEEIHALTDCSEKDLDFLHSVFQDQHLHTLLDLYDKINTKSSPQIRNPPSDAVQRAK
Capra_hircus              LDSLEEIHALTDCSEKDLDFLHSVFQDQHLHTLLDLYDKINTKSSPQIRNPPSDAVQRAK
Ovis_aries                LDSLEEIHALTDCSEKDLDFLHSVFQDQHLHTLLDLYDKINTKSSPQIRNPPSDAVQRAK
Equus_caballus            LDSLEEIHALTDCSEKDLDFLHSVFQDQHLHTLLDLYDKINTKSSPQIRNPPSDAVQRAK
Balaenoptera_musculus     LDSLEEIHALTDCSEKDLDFLHSVFQDQHLHTLLDLYDKINTKSSPQIRNPPSDAVQRAK
Rattus_norvegicus         LDSLEEIHALTDCSEKDLDFLHSVFQDQHLHTLLDLYDKINTKSSPQIRNPPSDAVQRAK
Microtus_oregoni          LDSLEEIHALTDCSEKDLDFLHSVFQDQHLHTLLDLYDKINTKSSPQIRNPPSDAVQRAK
Microtus_ochrogaster      LDSLEEIHALTDCSEKDLDFLHSVFQDQHLHTLLDLYDKINTKSSPQIRNPPSDAVQRAK
Pongo_abelii              LDSLEEIHALTDCSEKDLDFLHSVFQDQHLHTLLDLYDKINTKSSPQIRNPPSDAVQRAK
Zalophus_californianus    LDSLEEIHALTDCSEKDLDFLHSVFQDQHLHTLLDLYDKINTKSSPQIRNPPSDAVQRAK
Delphinapterus_leucas     LDSLEEIHALTDCSEKDLDFLHSVFQDQHLHTLLDLYDKINTKSSPQIRNPPSDAVQRAK
Mus_musculus              LDSLEEIHALTDCSEKDLDFLHSVFQDQHLHTLLDLYDKINTKSSPQIRNPPSDAVQRAK
```

```
                          410       420       430       440       450       460
Macaca_mulatta            EVLEEISCYPENNDAKELKRILTQPHFMALLQTHDVVAHEVYSDEALRVTPPPTSPYLNG
Macaca_fascicularis       EVLEEISCYPENNDAKELKRILTQPHFMALLQTHDVVAHEVYSDEALRVTPPPTSPYLNG
Echinops_telfairi         EVLEEISCYPENNDAKELKRILTQPHFMALLQTHDVVAHEVYSDEALRVTPPPTSPYLNG
Ornithorhynchus_anatinus  EVLEEISCYPENSDAKELKRILTQPHFMALLQTHDVVAHEVYSDEALRVTPPPTSPYLNG
Homo_sapiens              EVLEEISCYPENNDAKELKRILTQPHFMALLQTHDVVAHEVYSDEALRVTPPPTSPYLNG
Cavia_porcellus           EVLEEISCYPDNNDAKELKRILTQPHFMALLQTHDVVAHEVYSDEALRVTPPPTSPYLNG
Suricata_suricatta        EVLEEISCYPENNDAKELKRILTQPHFMALLQTHDVVAHEVYSDEALRVTPPPTSPYLNG
Macaca_nemestrina         EVLEEISCYPENNDAKELKRILTQPHFMALLQTHDVVAHEVYSDEALRVTPPPTSPYLNG
Tupaia_chinensis          EVLEEISCYPENNDAKELKRILTQPHFMALLQTHDVVAHEVYSDEALRVTPPPTSPYLNG
Bos_mutus                 EVLEEISCYPENNDAKELKRILTQPHFMALLQTHDVVAHEVYSDEALRVTPPPTSPYLNG
Pipistrellus_kuhlii       EVLEEISCYPENNDAKELKRILTQPHFMALLQTHDVVAHEVYSDEALRVTPPPTSPYLNG
Capra_hircus              EVLEEISCYPENNDAKELKRILTQPHFMALLQTHDVVAHEVYSDEALRVTPPPTSPYLNG
Ovis_aries                EVLEEISCYPENNDAKELKRILTQPHFMALLQTHDVVAHEVYSDEALRVTPPPTSPYLNG
Equus_caballus            EVLEEISCYPENNDAKELKRILTQPHFMALLQTHDVVAHEVYSDEALRVTPPPTSPYLNG
Balaenoptera_musculus     EVLEEISCYPENNDAKELKRILTQPHFMALLQTHDVVAHEVYSDEALRVTPPPTSPYLNG
Rattus_norvegicus         EVLEEISCYPENNDAKELKRILTQPHFMALLQTHDVVAHEVYSDEALRVTPPPTSPYLNG
Microtus_oregoni          EVLEEISCYPENNDAKELKRILTQPHFMALLQTHDVVAHEVYSDEALRVTPPPTSPYLNG
Microtus_ochrogaster      EVLEEISCYPENNDAKELKRILTQPHFMALLQTHDVVAHEVYSDEALRVTPPPTSPYLNG
Pongo_abelii              EVLEEISCYPENNDAKELKRILTQPHFMALLQTHDVVAHEVYSDEALRVTPPPTSPYLNG
Zalophus_californianus    EVLEEISCYPENNDAKELKRILTQPHFMALLQTHDVVAHEVYSDEALRVTPPPTSPYLNG
Delphinapterus_leucas     EVLEEISCYPENNDAKELKRILTQPHFMALLQTHDVVAHEVYSDEALRVTPPPTSPYLNG
Mus_musculus              EVLEEISCYPENNDAKELKRILTQPHFMALLQTHDVVAHEVYSDEALRVTPPPTSPYLNG
```

```
                          470       480       490       500       510       520
Macaca_mulatta            DSPESANGDMDMENVTRVRLVQFQKNTDEPMGITLKMNELNHCIVARIMHGGMIHRQGTL
Macaca_fascicularis       DSPESANGDMDMENVTRVRLVQFQKNTDEPMGITLKMNELNHCIVARIMHGGMIHRQGTL
Echinops_telfairi         DSPESANGDMDMENVTRVRLVQFQKNTDEPMGITLKMNELNHCIVARIMHGGMIHRQGTL
Ornithorhynchus_anatinus  DSPESANGDMDMENVTRVRLVQFQKNTDEPMGITLKMNELNHCIVARIMHGGMIHRQGTL
Homo_sapiens              DSPESANGDMDMENVTRVRLVQFQKNTDEPMGITLKMNELNHCIVARIMHGGMIHRQGTL
Cavia_porcellus           DSPESANGDMDMENVTRVRLVQFQKNTDEPMGITLKMNELNHCIVARIMHGGMIHRQGTL
Suricata_suricatta        DSPESANGDMDMENVTRVRLVQFQKNTDEPMGITLKMNELNHCIVARIMHGGMIHRQGTL
Macaca_nemestrina         DSPESANGDMDMENVTRVRLVQFQKNTDEPMGITLKMNELNHCIVARIMHGGMIHRQGTL
Tupaia_chinensis          DSPESANGDMDMENVTRVRLVQFQKNTDEPMGITLKMNELNHCIVARIMHGGMIHRQGTL
Bos_mutus                 DSPESANGDMDMENVTRVRLVQFQKNTDEPMGITLKMNELNHCIVARIMHGGMIHRQGTL
Pipistrellus_kuhlii       DSPESANGDMDMENVTRVRLVQFQKNTDEPMGITLKMNELNHCIVARIMHGGMIHRQGTL
Capra_hircus              DSPESANGDMDMENVTRVRLVQFQKNTDEPMGITLKMNELNHCIVARIMHGGMIHRQGTL
Ovis_aries                DSPESANGDMDMENVTRVRLVQFQKNTDEPMGITLKMNELNHCIVARIMHGGMIHRQGTL
Equus_caballus            DSPESANGDMDMENVTRVRLVQFQKNTDEPMGITLKMNELNHCIVARIMHGGMIHRQGTL
Balaenoptera_musculus     DSPESANGDMDMENVTRVRLVQFQKNTDEPMGITLKMNELNHCIVARIMHGGMIHRQGTL
Rattus_norvegicus         DSPESANGDMDMENVTRVRLVQFQKNTDEPMGITLKMNELNHCIVARIMHGGMIHRQGTL
Microtus_oregoni          DSPESANGDMDMENVTRVRLVQFQKNTDEPMGITLKMNELNHCIVARIMHGGMIHRQGTL
Microtus_ochrogaster      DSPESANGDMDMENVTRVRLVQFQKNTDEPMGITLKMNELNHCIVARIMHGGMIHRQGTL
Pongo_abelii              DSPESANGDMDMENVTRVRLVQFQKNTDEPMGITLKMNELNHCIVARIMHGGMIHRQGTL
Zalophus_californianus    DSPESANGDMDMENVTRVRLVQFQKNTDEPMGITLKMNELNHCIVARIMHGGMIHRQGTL
Delphinapterus_leucas     DSPESANGDMDMENVTRVRLVQFQKNTDEPMGITLKMNELNHCIVARIMHGGMIHRQGTL
Mus_musculus              DSPESANGDMDMENVTRVRLVQFQKNTDEPMGITLKMNELNHCIVARIMHGGMIHRQGTL
```

```
                          530       540       550       560       570       580
Macaca_mulatta            HVGDEIREINGISVANQTVEQLQKMLREMRGSITFKIVPSYRTQSSSCERDSPSTSRQSP
Macaca_fascicularis       HVGDEIREINGISVANQTVEQLQKMLREMRGSITFKIVPSYRTQSSSCERDSPSTSRQSP
Echinops_telfairi         HVGDEIREINGISVANQTVEQLQKMLREMRGSITFKIVPSYRTQSSSCERDSPSTSRQSP
Ornithorhynchus_anatinus  HVGDEIREINGISVANQTVEQLQKMLREMRGSITFKIVPSYRTQSSSCERDSPSTSRQSP
Homo_sapiens              HVGDEIREINGISVANQTVEQLQKMLREMRGSITFKIVPSYRTQSSSCERDSPSTSRQSP
Cavia_porcellus           HVGDEIREINGISVANQTVEQLQKMLREMRGSITFKIVPSYRTQSSSCERDSPSTSRQSP
Suricata_suricatta        HVGDEIREINGISVANQTVEQLQKMLREMRGSITFKIVPSYRTQSSSCERDSPSTSRQSP
Macaca_nemestrina         HVGDEIREINGISVANQTVEQLQKMLREMRGSITFKIVPSYRTQSSSCERDSPSTSRQSP
Tupaia_chinensis          HVGDEIREINGISVANQTVEQLQKMLREMRGSITFKIVPSYRTQSSSCERDSPSTSRQSP
Bos_mutus                 HVGDEIREINGISVANQTVEQLQKMLREMRGSITFKIVPSYRTQSSSCERDSPSTSRQSP
Pipistrellus_kuhlii       HVGDEIREINGISVANQTVEQLQKMLREMRGSITFKIVPSYRTQSSSCERDSPSTSRQSP
Capra_hircus              HVGDEIREINGISVANQTVEQLQKMLREMRGSITFKIVPSYRTQSSSCERDSPSTSRQSP
Ovis_aries                HVGDEIREINGISVANQTVEQLQKMLREMRGSITFKIVPSYRTQSSSCERDSPSTSRQSP
Equus_caballus            HVGDEIREINGISVANQTVEQLQKMLREMRGSITFKIVPSYRTQSSSCERDSPSTSRQSP
Balaenoptera_musculus     HVGDEIREINGISVANQTVEQLQKMLREMRGSITFKIVPSYRTQSSSCERDSPSTSRQSP
Rattus_norvegicus         HVGDEIREINGISVANQTVEQLQKMLREMRGSITFKIVPSYRTQSSSCERDSPSTSRQSP
Microtus_oregoni          HVGDEIREINGISVANQTVEQLQKMLREMRGSITFKIVPSYRTQSSSCERDSPSTSRQSP
Microtus_ochrogaster      HVGDEIREINGISVANQTVEQLQKMLREMRGSITFKIVPSYRTQSSSCERDSPSTSRQSP
Pongo_abelii              HVGDEIREINGISVANQTVEQLQKMLREMRGSITFKIVPSYRTQSSSCERDSPSTSRQSP
Zalophus_californianus    HVGDEIREINGISVANQTVEQLQKMLREMRGSITFKIVPSYRTQSSSCERESPSTSRQSP
Delphinapterus_leucas     HVGDEIREINGISVANQTVEQLQKMLREMRGSITFKIVPSYRTQSSSCERDSPSTSRQSP
Mus_musculus              HVGDEIREINGISVANQTVEQLQKMLREMRGSITFKIVPSYRTQSSSCERDSPSTSRQSP
```

```
                          590       600       610       620       630       640
Macaca_mulatta            ANGHSSTNNSVSDLPSTTQPKGRQIYVRAQFEYDPAKDDLIPCKEAGIRFRVGDIIQIIS
Macaca_fascicularis       ANGHSSTNNSVSDLPSTTQPKGRQIYVRAQFEYDPAKDDLIPCKEAGIRFRVGDIIQIIS
Echinops_telfairi         ANGHSSTNNSVSDLPSTTQPKGRQIYVRAQFEYDPAKDDLIPCKEAGIRFRVGDIIQIIS
Ornithorhynchus_anatinus  ANGPSSTNNSVSDLPSTTQPKGRQIYVRAQFEYDPAKDDLIPCKEAGIRFRVGDIIQIIS
Homo_sapiens              ANGHSSTNNSVSDLPSTTQPKGRQIYVRAQFEYDPAKDDLIPCKEAGIRFRVGDIIQIIS
Cavia_porcellus           ANGHSSTNNSVSDLPSTTQPKGRQIYVRAQFEYDPAKDDLIPCKEAGIRFRVGDIIQIIS
Suricata_suricatta        ANGHSSTNNSVSDLPSTTQPKGRQIYVRAQFEYDPAKDDLIPCKEAGIRFRVGDIIQIIS
Macaca_nemestrina         ANGHSSTNNSVSDLPSTTQPKGRQIYVRAQFEYDPAKDDLIPCKEAGIRFRVGDIIQIIS
Tupaia_chinensis          ANGHSSTNNSVSDLPSTTQPKGRQIYVRAQFEYDPAKDDLIPCKEAGIRFRVGDIIQIIS
Bos_mutus                 ANGHSSTNNSVSDLPSTTQPKGRQIYVRAQFEYDPAKDDLIPCKEAGIRFRVGDVIQIIS
Pipistrellus_kuhlii       ANGHSSTNNSVSDLPSTTQPKGRQIYVRAQFEYDPAKDDLIPCKEAGIRFRVGDVIQIIS
Capra_hircus              ANGHSSTNNSVSDLPSTTQPKGRQIYVRAQFEYDPAKDDLIPCKEAGIRFRVGDIIQIIS
Ovis_aries                ANGHSSTNNSVSDLPSTTQPKGRQIYVRAQFEYDPAKDDLIPCKEAGIRFRVGDIIQIIS
Equus_caballus            ANGHSSTNNSVSDLPSTTQPKGRQIYVRAQFEYDPAKDDLIPCKEAGIRFRVGDIIQIIS
Balaenoptera_musculus     ANGHSSTNNSVSDLPSTTQPKGRQIYVRAQFEYDPAKDDLIPCKEAGIRFRVGDIIQIIS
Rattus_norvegicus         ANGHSSTNNSVSDLPSTTQPKGRQIYVRAQFEYDPAKDDLIPCKEAGIRFRVGDIIQIIS
Microtus_oregoni          ANGHSSTNNTVSDLPSTTQPKGRQIYVRAQFEYDPAKDDLIPCKEAGIRFRVSDIIQIIS
Microtus_ochrogaster      ANGHSSTNNSVSDLPSTTQPKGRQIYVRAQFEYDPAKDDLIPCKEAGIRFRVGDIIQIIS
Pongo_abelii              ANGHSSTNNSVSDLPSTTQPKGRQIYVRAQFEYDPAKDDLIPCKEAGIRFRVGDIIQIIS
Zalophus_californianus    ANGHSSTNNSVSDLPSTTQPKGRQIYVRAQFEYDPAKDDLIPCKEAGIRFRVGDIIQIIS
Delphinapterus_leucas     ANGHSSTNNSVSDLPSTTQPKGRQIYVRAQFEYDPAKDDLIPCKEAGIRFRVGDIIQIIS
Mus_musculus              ANGHSSTNNSVSDLPSTTQPKGRQIYVRAQFEYDPAKDDLIPCKEAGIRFRVGDIIQIIS
```

```
                          650       660       670       680       690       700
Macaca_mulatta            KDDHNWWQGKLENSKNGTAGLIPSPELQEWRVACIAMEKTKQEQQASCTWFGKKKKQYKD
Macaca_fascicularis       KDDHNWWQGKLENSKNGTAGLIPSPELQEWRVACIAMEKTKQEQQASCTWFGKKKKQYKD
Echinops_telfairi         KDDHNWWQGKLENSKNGTAGLIPSPELQEWRVACIAMEKTKQEQQASCTWFGKKKKQYKD
Ornithorhynchus_anatinus  KDDHNWWQGKLENSKNGTAGLIPSPELQEWRVACIAMEKTKQEQQASCTWFGKKKKQYKD
Homo_sapiens              KDDHNWWQGKLENSKNGTAGLIPSPELQEWRVACIAMEKTKQEQQASCTWFGKKKKQYKD
Cavia_porcellus           KDDHNWWQGKLENSKNGTAGLIPSPELQEWRVACIAMEKTKQEQQASCTWFGKKKKQYKD
Suricata_suricatta        KDDHNWWQGKLENSKNGTAGLIPSPELQEWRVACIAMEKTKQEQQASCTWFGKKKKQYKD
Macaca_nemestrina         KDDHNWWQGKLENSKNGTAGLIPSPELQEWRVACIAMEKTKQEQQASCTWFGKKKKQYKD
Tupaia_chinensis          KDDHNWWQGKLENSKNGTAGLIPSPELQEWRVACIAMEKTKQEQQASCTWFGKKKKQYKD
Bos_mutus                 KDDHNWWQGKLENSKNGAAGLIPSPELQEWRVACIAMEKTKQEQQASCTWFGKKKKQYKD
Pipistrellus_kuhlii       KDDHNWWQGKLENSKNGTAGLIPSPELQEWRVACIAMEKTKQEQQASCTWFGKKKKQYKD
Capra_hircus              KDDHNWWQGKLENSKNGTAGLIPSPELQEWRVACIAMEKTKQEQQASCTWFGKKKKQYKD
Ovis_aries                KDDHNWWQGKLENSKNGTAGLIPSPELQEWRVACIAMEKTKQEQQASCTWFGKKKKQYKD
Equus_caballus            KDDHNWWQGKLENSKNGTAGLIPSPELQEWRVACIAMEKTKQEQQASCTWFGKKKKQYKD
Balaenoptera_musculus     KDDHNWWQGKLENSKNGTAGLIPSPELQEWRVACIAMEKTKQEQQASCTWFGKKKKQYKD
Rattus_norvegicus         KDDHNWWQGKLENSKNGTAGLIPSPELQEWRVACIAMEKTKQEQQASCTWFGKKKKQYKD
Microtus_oregoni          KDDHNWWQGKLENSKNGTAGLIPSPELQEWRVACIAMEKTKQEQQASCTWFGKKKKQYKD
Microtus_ochrogaster      KDDHNWWQGKLENSKNGTAGLIPSPELQEWRVACIAMEKTKQEQQASCTWFGKKKKQYKD
Pongo_abelii              KDDHNWWQGKLENSKNGTAGLIPSPELQER.VACIAMEKTKQEQQASCTWFGKKKKQYKD
Zalophus_californianus    KDDHNWWQGKLENSKNGTAGLIPSPELQEWRVACIAMEKTKQEQQASCTWFGKKKKQYKD
Delphinapterus_leucas     KDDHNWWQGKLENSKNGTAGLIPSPELQEWRVACIAMEKTKQEQQASCTWFGKKKKQYKD
Mus_musculus              KDDHNWWQGKLENSKNGTAGLIPSPELQEWRVACIAMEKTKQEQQASCTWFGKKKKQYKD
```

```
                              710       720       730       740       750       760
                              .         .         .         .         .         .
Macaca_mulatta            KYLAKHN.....ADLVTYEEVVKLPAFKRKTLVLLGAHGVGRRHIKNTLITKHPDRFAYP
Macaca_fascicularis       KYLAKHN.....ADLVTYEEVVKLPAFKRKTLVLLGAHGVGRRHIKNTLITKHPDRFAYP
Echinops_telfairi         KYLAKHN.....ADLVTYEEVVKLPAFKRKTLVLLGAHGVGRRHIKNTLITKHPDRFAYP
Ornithorhynchus_anatinus  KYLAKHN.....ADLVTYEEVVKLPAFKRKTLVLLGAHGVGRRHIKNTLITKHPDRFAYP
Homo_sapiens              KYLAKHN.....ADLVTYEEVVKLPAFKRKTLVLLGAHGVGRRHIKNTLITKHPDRFAYP
Cavia_porcellus           KYLAKHN.....ADLVTYEEVVKLPAFKRKTLVLLGAHGVGRRHIKNTLITKHPDRFAYP
Suricata_suricatta        KYLAKHN.....ADLVTYEEVVKLPAFKRKTLVLLGAHGVGRRHIKNTLITKHPDRFAYP
Macaca_nemestrina         KYLAKHN.....ADLVTYEEVVKLPAFKRKTLVLLGAHGVGRRHIKNTLITKHPDRFAYP
Tupaia_chinensis          KYLAKHN.....ADLVTYEEVVKLPAFKRKTLVLLGAHGVGRRHIKNTLITKHPDRFAYP
Bos_mutus                 KYLAKHN.....ADLVTYEEVVKLPAFKRKTLVLLGAHGVGRRHIKNTLITKHPDRFAYP
Pipistrellus_kuhlii       KYLAKHN.....ADLVTYEEVVKLPAFKRKTLVLLGAHGVGRRHIKNTLITKHPDRFAYP
Capra_hircus              KYLAKHN.....ADLVTYEEVVKLPAFKRKTLVLLGAHGVGRRHIKNTLITKHPDRFAYP
Ovis_aries                KYLAKHNAVFDQLDLVTYEEVVKLPAFKRKTLVLLGAHGVGRRHIKNTLITKHPDRFAYP
Equus_caballus            KYLAKHNAVFDQLDLVTYEEVVKLPAFKRKTLVLLGAHGVGRRHIKNTLITKHPDRFAYP
Balaenoptera_musculus     KYLAKHN.....ADLVTYEEVVKLTAFKRKTLVLLGAHGVGRRHIKNTLITKHPDRFAYP
Rattus_norvegicus         KYLAKHN.....ADLVTYEEVVKLPAFKRKTLVLLGAHGVGRRHIKNTLITKHPERFAYP
Microtus_oregoni          KYLAKHN.....ADLVTYEEVVKLPAFKRKTLVLLGAHGVGRRHIKNTLITKHPDRFAYP
Microtus_ochrogaster      KYLAKHN.....ADLVTYEEVVKLPAFKRKTLVLLGAHGVGRRHIKNTLITKHPDRFAYP
Pongo_abelii              KYLAKHNAVFDQLDLVTYEEVVKLPAFKRKTLVLLGAHGVGRRHIKNTLITKHPDRFAYP
Zalophus_californianus    KYLAKHNAVFDQLDLVTYEEVVKLPAFKRKTLVLLGAHGVGRRHIKNTLITKHPDRFAYP
Delphinapterus_leucas     KYLAKHNAVFDQLDLVTYEEVVKLTAFKRKTLVLLGAHGVGRRHIKNTLITKHPDRFAYP
Mus_musculus              KYLAKHNAVFDQLDLVTYEEVVKLPAFKRKTLVLLGAHGVGRRHIKNTLITKHPDRFAYP
```

```
                                    770       780       790       800       810       820
                                    .         .         .         .         .         .
Macaca_mulatta            IPHTTRPPKKDEENGKNYYFVSHDQMMQDISNNEYLEYGSHEDAMYGTKLETIRKIHEQG
Macaca_fascicularis       IPHTTRPPKKDEENGKNYYFVSHDQMMQDISNNEYLEYGSHEDAMYGTKLETIRKIHEQG
Echinops_telfairi         IPHTTRPPKKDEENGKNYYFVSHDQMMQDISNNEYLEYGSHEDAMYGTKLETIRKLHEQG
Ornithorhynchus_anatinus  IPHTTRPPKKDEENGKNYYFVSHDQMMQDISNNEYLEYGSHEDAMYGTKLETIRKIHEQG
Homo_sapiens              IPHTTRPPKKDEENGKNYYFVSHDQMMQDISNNEYLEYGSHEDAMYGTKLETIRKIHEQG
Cavia_porcellus           IPHTTRPPKKDEENGKNYYFVSHDQMMQDISNNEYLEYGSHEDAMYGTKLETIRKIHEQG
Suricata_suricatta        IPHTTRPPKKDEENGKNYYFVSHDQMMQDISNNEYLEYGSHEDAMYGTKLETIRKIHEQG
Macaca_nemestrina         IPHTTRPPKKDEENGKNYYFVSHDQMMQDISNNEYLEYGSHEDAMYGTKLETIRKIHEQG
Tupaia_chinensis          IPHTTRPPKKDEENGKNYYFVSHDQMMQDISNNEYLEYGSHEDAMYGTKLETIRKIHEQG
Bos_mutus                 IPHTTRPPKKDEENGKNYYFVSHDQMMQDISNNEYLEYGSHEDAMYGTKLETIRKIHEQG
Pipistrellus_kuhlii       IPHTTRPPKKDEENGKNYYFVSHDQMMQDISNNEYLEYGSHEDAMYGTKLETIRKIHEQG
Capra_hircus              IPHTTRPPKKDEENGKNYYFVSHDQMMQDISNNEYLEYGSHEDAMYGTKLETIRKIHEQG
Ovis_aries                IPHTTRPPKKDEENGKNYYFVSHDQMMQDISNNEYLEYGSHEDAMYGTKLETIRKIHEQG
Equus_caballus            IPHTTRPPKKDEENGKNYYFVSHDQMMQDISNNEYLEYGSHEDAMYGTKLETIRKIHEQG
Balaenoptera_musculus     IPHTTRPPKKDEENGKNYYFVSHDQMMQDISNNEYLEYGSHEDAMYGTKLETIRKIHEQG
Rattus_norvegicus         IPHTTRPPKKDEENGKNYYFVSHDQMMQDISNNEYLEYGSHEDAMYGTKLETIRKIHEQG
Microtus_oregoni          IPHTTRPPKKDEENGKNYYFVSHDQMMQDISNNEYLEYGSHEDAMYGTKLETIRKIHEQG
Microtus_ochrogaster      IPHTTRPPKKDEENGKNYYFVSHDQMMQDISNNEYLEYGSHEDAMYGTKLETIRKIHEQG
Pongo_abelii              IPHTTRPPKKDEENGKNYYFVSHDQMMQDISNNEYLEYGSHEDAMYGTKLETIRKIHEQG
Zalophus_californianus    IPHTTRPPKKDEENGKNYYFVSHDQMMQDISNNEYLEYGSHEDAMYGTKLETIRKIHEQG
Delphinapterus_leucas     IPHTTRPPKKDEENGKNYYFVSHDQMMQDISNNEYLEYGSHEDAMYGTKLETIRKIHEQG
Mus_musculus              IPHTTRPPKKDEENGKNYYFVSHDQMMQDISNNEYLEYGSHEDAMYGTKLETIRKIHEQG
```

```
                                    830       840       850       860       870       880
                                    .         .         .         .         .         .
Macaca_mulatta            LIAILDVEPQALKVLRTAEFAPFVVFIAAPTITPGLNEDESLQRLQKESDILQRTYAHYF
Macaca_fascicularis       LIAILDVEPQALKVLRTAEFAPFVVFIAAPTITPGLNEDESLQRLQKESDILQRTYAHYF
Echinops_telfairi         LIAILDVEPQALKVLRTAEFAPFVVFIAAPTITPGLNEDESLQRLQKESDILQRTYAHYF
Ornithorhynchus_anatinus  LIAILDVEPQALKVLRTAEFAPFVVFIAAPTITPGINEDESLQRLQKESEILQRTYAHYF
Homo_sapiens              LIAILDVEPQALKVLRTAEFAPFVVFIAAPTITPGLNEDESLQRLQKESDILQRTYAHYF
Cavia_porcellus           LIAILDVEPQALKVLRTAEFAPFVVFIAAPTITPGLNEDESLQRLQKESDILQRTYAHYF
Suricata_suricatta        LIAILDVEPQALKVLRTAEFAPFVVFIAAPTITPGLNEDESLQRLQKESDILQRTYAHYF
Macaca_nemestrina         LIAILDVEPQALKVLRTAEFAPFVVFIAAPTITPGLNEDESLQRLQKESDILQRTYAHYF
Tupaia_chinensis          LIAILDVEPQALKVLRTAEFAPFVVFIAAPTITPGLNEDESLQRLQKESDILQRTYAHYF
Bos_mutus                 LIAILDVEPQALKVLRTAEFAPFVVFIAAPTITPGLNEDESLQRLQKESDILQRTYAHYF
Pipistrellus_kuhlii       LIAILDVEPQALKVLRTAEFAPFVVFIAAPTITPGLNEDESLQRLQKESDILQRTYAHYF
Capra_hircus              LIAILDVEPQALKVLRTAEFAPFVVFIAAPTITPGLNEDESLQRLQKESDILQRTYAHYF
Ovis_aries                LIAILDVEPQALKVLRTAEFAPFVVFIAAPTITPGLNEDESLQRLQKESDILQRTYAHYF
Equus_caballus            LIAILDVEPQALKVLRTAEFAPFVVFIAAPTITPGLNEDESLQRLQKESDILQRTYAHYF
Balaenoptera_musculus     LIAILDVEPQALKVLRTAEFAPFVVFIAAPTITPGLNEDESLQRLQKESDILQRTYAHYF
Rattus_norvegicus         LIAILDVEPQALKVLRTAEFAPFVVFIAAPTITPGLNEDESLQRLQKESDVLQRTYAHYF
Microtus_oregoni          LIAILDVEPQALKVLRTAEFAPFVVFIAAPTITPGLNEDESLQRLQKESDVLQRTYAHYF
Microtus_ochrogaster      LIAILDVEPQALKVLRTAEFAPFVVFIAAPTITPGLNEDESLQRLQKESDVLQRTYAHYF
Pongo_abelii              LIAILDVEPQALKVLRTAEFAPFVVFIAAPTITPGLNEDESLQRLQKESDILQRTYAHYF
Zalophus_californianus    LIAILDVEPQALKVLRTAEFAPFVVFIAAPTITPGLNEDESLQRLQKESDILQRTYAHYF
Delphinapterus_leucas     LIAILDVEPQALKVLRTAEFAPFVVFIAAPTITPGLNEDESLQRLQKESDILQRTYAHYF
Mus_musculus              LIAILDVEPQALKVLRTAEFAPFVVFIAAPTITPGLNEDESLQRLQKESDVLQRTYAHYF
```

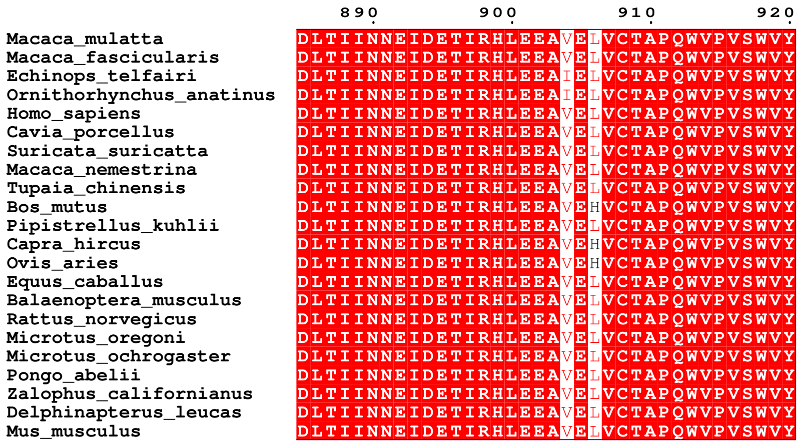

Supplemental figure 2. Comparative amino acid sequence alignment of CASK across various species.

Homo sapiens, NP_003679.2; *Pongo abelii*, PNJ69644.1; *Macaca mulatta*, EHH30653.1; *Macaca fascicularis*, EHH60822.1; *Macaca nemestrina*, XP_011708592.1; *Rattus norvegicus*, XP_038955489.1; *Mus musculus*, NP_001271432.1; *Microtus ochrogaster,* XP_005352928.1; *Microtus oregoni*, XP_041501183.1; *Cavia porcellus*, XP_003469478.2; *Suricata suricatta*, XP_029785267.1; *Tupaia chinensis*, XP_027627819.1; *Ovis aries*, XP_011961918.2; *Capra hircus*, XP_017900064.1; *Bos mutus*, XP_005889513.1; *Equus caballus*, XP_014584296.2; *Zalophus californianus*, XP_027463799.1; *Delphinapterus leucas*, XP_022412132.1; *Balaenoptera musculus*, XP_036695396.1; *Echinops telfairi*, XP_004714633.1; *Pipistrellus kuhlii*, KAF6294542.1; *Ornithorhynchus anatinus*, XP_007665435.1
