# Supplementary material for: Case Report: Identification of a novel CASK missense variant in a Chinese family with MICPCH
Source: Front Genet. 2022 Aug 25;13:933785. doi: 10.3389/fgene.2022.933785 (PMC9452731; doi:10.3389/fgene.2022.933785)
Supplement: Supplementary file 7 [file Table5.DOCX]

| **Table 1. Summary of the intragenic nucleotide changes in *CASK* and phenotypic features of the male patients** | | | | | | | |  |
| --- | --- | --- | --- | --- | --- | --- | --- | --- |
| **Number** | **Gender** | **Age** | **Clinical features** | **Mutation*** | **Detection** | **Inheritance** | **Previous report** | **References** |
| 1 | M | 4y | Ohtahara syndrome and cerebellar hypoplasia | c.1A>G (p.M1V) | WES | de novo | patient 2 in PMID: 22709267 | 7 |
| 2 | M | 4y4m | Microcephaly (-4.9SD) | c.317G>C (p.R106P) | DS | de novo | Patient 22 in PMID: 28783747 | 18 |
| 3 | M | 4y | Infantile spasms | c.437G>A (p.C146Y) | WES | de novo | patient in PMID: 31957018 | 27 |
| 4 | M | NA | Intellectual disability | c.490G>A (p.G164R) | WES | Maternal | patient in PMID: 31906484 | 28 |
| 5 | M | NA | Severe to profound mental retardation, seizure, autistic | c.802T>C (p.Y268H) | DS | NA | patient in PMID: 20029458 | 9 |
| 6 | M | NA | FG syndrome | c.979G>A (p.E327K) | WES | NA | patient in PMID: 29758562 | 29 |
| 7 | M | 4m | Microcephaly (-4SD), severe developmental delay | c.1061T>C (p.L354P) | DS | NA | Patient 16 in PMID: 23165780 | 5 |
| 8 | M | NA | Intellectual disability, autism spectrum disorder/autistic traits, epilepsy and hypotonia | c.1159T>C (p.Y387H) | WES | de novo | patient in PMID: 31144778 | 30 |
| 9 | M | NA | Profound mental retardation | c.1186C>T(p.P396S) | DS | NA | patient in PMID: 20029458 | 9 |
| 10 | M | NA | Microphthalmia, anophthalmia and coloboma disease | c.1289G>A (p.R430H) | WES | Maternal | patient in PMID: 32799327 | 31 |
| 11 | M | 3y | Microcephaly, developmental delay, mild ID, short stature, umbilical herniation, poor verbal communication | c.1424G>T (S475I) | WES | Maternal | patient in PMID: 28944139 | 32 |
| 12 | M | 20-40y | Severe intellectual disability, seizures, hypertonia | c.1465C>T/p.(R489W) | WES | Maternal and de novo | patient 1 and patient 2 in PMID: 33090494 | 33 |
| 13 | M | 3.5y | Neurodevelopmental disorder | c.1521G>T (p.M507I) | WES | Maternal | patient 3 in PMID: 33090494 | 33 |
| 14 | M | NA | severe intellectual disability, seizures, microcephaly, abnormal hearing, hypertonia, facial dysmorphism | c.1562G>T (p.G521V) | Gene panel or WES | Maternal | patient in PMID: 33090494 | 33 |
| 15 | M | NA | Epileptic encephalopathy | p.R584X | Gene panel | de novo | patient in PMID: 27652284 | 34 |
| 16 | M | NA | Multiple congenital anomalies, developmental disorder and microcephaly | c.1874C>T (p.P625L) | NA | de novo | patient in PMID: 28135719 | 35 |
| 17 | M | 8y, 4y | Microcephaly, intellectual disability, ataxia, gait disorder and unable to speak | c.1882 G > C (p.D628H) | WES | Maternal | patient in this study |  |
| 18 | M | 4.9y | Epilepsy and autism spectrum disorder | c.1922G>A (p.R641K) | WES | Maternal | patient in PMID: 31139143 | 36 |
| 19 | M | 3y | Microcephaly, MICPCH, optic nerve hypoplasia | c.2018C>T (p.P673L) | WES | NA | patient in PMID: 31425583 | 37 |
| 20 | M | 32y | Developmental delay, hearing loss and dilated cardiomyopathy | c.2126A>G (p.K709R) | WES | Maternal | patient in PMID: 27173948 | 38 |
| 21 | M | NA | Mental retardation, nystagmus | c.2129A>G (p.D710G) | Gene panel | NA | patient in PMID: 19377476 | 11 |
| 22 | M | 14-19y | Mental retardation, nystagmus and microcephaly | c.2183A>G (p.Y728C) | DS | Maternal | two patients in Family V in PMID: 20029458 | 9 |
| 23 | M |  | MRI at 24 + 5 weeks: agenesis of corpus callosum, brain cyst type IIC, subcortical heterotopia and bilateral frontal polymicrogyria | c.2392A>T (p.N798Y) | DS | NA | patient 5 in PMID: 33090494 | 33 |
| 24 | M | 2m | Neonatal hypotonia, hyperkinesia, and postnatal microcephaly with occipito frontal circumference, mild coarse facial features, seizures | c.2546T>C (p.V849A) | WES | NA | patient in PMID: 31736593 | 39 |
| 25 | M | NA | Mild mental retardation, nystagmus, seizure | c.2756T>C (p.W919R) | DS | NA | patient in PMID: 20029458 | 9 |
| *Reference sequence NM_003688.3 for nucleotide, NP_003679.2 for protein. | | | | | | | |  |
